# correctKin: an optimized method to infer relatedness up to the 4th degree from low-coverage ancient human genomes

**DOI:** 10.1186/s13059-023-02882-4

**Published:** 2023-02-28

**Authors:** Emil Nyerki, Tibor Kalmár, Oszkár Schütz, Rui M. Lima, Endre Neparáczki, Tibor Török, Zoltán Maróti

**Affiliations:** 1 Department of Pediatrics, University of Szeged Albert Szent-Györgyi Medical Center Faculty of Medicine, Szeged, Hungary; 2 Department of Archaeogenetics, Institute of Hungarian Research, Budapest, Hungary; 3 Department of Genetics, University of Szeged, Szeged, Hungary; 4 Institute of Plant Biology, Biological Research Centre, Szeged, Hungary

**Keywords:** Kinship, Genomics, Low coverage, Ancient DNA, Forensic

## Abstract

Kinship analysis from very low-coverage ancient sequences has been possible up to the second degree with large uncertainties. We propose a new, accurate, and fast method, correctKin, to estimate the kinship coefficient and the confidence interval using low-coverage ancient data. We perform simulations and also validate correctKin on experimental modern and ancient data with widely different genome coverages (0.12×–11.9×) using samples with known family relations and known/unknown population structure. Based on our results, correctKin allows for the reliable identification of relatedness up to the 4th degree from variable/low-coverage ancient or badly degraded forensic whole genome sequencing data.

**Supplementary Information:**

The online version contains supplementary material available at 10.1186/s13059-023-02882-4.

## Background

Kinship analysis is a method for determination of the familial relationship between individuals from genome data. The kinship coefficient is defined as the probability that two homologous alleles drawn from each of two individuals are the result of identity by descent (IBD). This is a classic measurement of relatedness [1, 2]. Several algorithms have been developed to perform kinship analysis [3] including GERMLINE [4], fastIBD [5], GRAB [6], and ANGSD [7]. These are based on different strategies and metrics of IBD segments for calculating relatedness from microarray or WGS data. Distinguishing IBD which represents familial relatedness from identity-by-state (IBS) that represents population relatedness is difficult as both result in genetic similarity based on shared alleles. Despite the biological variation in IBD sharing due to the outcome of the stochastic nature of recombination and segregation during meiosis in gametogenesis, it is possible to infer kinship up to the 5–6th degree of relatedness from microarray or deeply typed WGS data. Achieving such a high level of certainty also requires an appropriate set of reference data (except for methods like KING [8] or IBIS [9]), and clever algorithms that account for biological variations resulting from familial (IBD) and population relatedness (IBS).

Recently huge genomic datasets have been generated from ancient samples in order to uncover the genetic relations of ancient and modern populations. From this data, it is also of high interest to study the family organization of ancient populations. However, analyzing ancient DNA (aDNA) poses additional difficulties due to the widely different but generally low genome coverage and postmortem damage (PMD) observed in these samples. The aDNA databases usually contain sequence data between 0.05 and 3× average genome coverage [10–12], since the sequencing of ancient samples with low endogenous DNA content is still challenging and costly. Differences in coverage and only partial overlap of genetic markers between samples can lead to significant bias when comparing the frequencies and genotype likelihoods of genetic variants, leading to uncertainties of the inferred genotype probabilities. An additional problem in the analysis of ancient data is that in most cases there is limited or no information on the appropriate reference population data to distinguish IBD from IBS.

In the present study, we wanted to address the difficulties of low-coverage aDNA data and dissect the main factors that affect kinship calculations. To overcome typing bias, random sampling of one allele per site (pseudo-haploid calling) was used successfully in aDNA studies [13–22]. In order to compare diploid and pseudo-haploid datasets, heterozygous alleles of diploid data need to be random pseudo-haploidized (RpsH) by randomly assigning heterozygote alleles as either homozygote reference (REF) or homozygote alternative (ALT). Although rare alleles can offer significant improvement in some kinship calculation methods when analyzing high-quality WGS data, genotype calling from the whole human genome could lead to excessive, variable amounts of false positive calls from low-coverage, degraded aDNA datasets. To minimize this bias, we restricted our analysis to the already known biallelic, high-frequency, and population-informative SNPs of the V42.2 1240K Allen Ancient DNA Resource (AADR) dataset [23]. To address the issue of unknown reference populations, we used the PC-Relate algorithm [24]. In the presence of unspecified population structure, this algorithm proposes a principal component-based, model-free approach for estimating kinship coefficients and IBD sharing probabilities. We applied a combination of techniques to mitigate genotyping uncertainties and tested their effects and limitations on kinship analysis of low-coverage ancient sequences. We used simulation to downsample fully genotyped real NGS data to examine the effect of partial marker overlap between samples and we also explored the effect of reference population choice on the kinship coefficient calculation. Based on our results, we developed a new computational approach which can reliably calculate corrected kinship coefficient from poorly genotyped data.

Here we offer guidelines and a list of the necessary bioinformatics tools required to calculate the corrected kinship coefficient. These guidelines overcome the technical limitations of generally low genome coverage, postmortem damage, genotyping uncertainties, and the partial overlapping of genetic markers between samples. As a proof of concept, we validated our proposed methodology on both experimental modern and ancient data with widely different genome coverages, using samples with known family relations and known or unknown population structure.

## Results

To use marker counts similar to aDNA data, all modern dataset were downsampled to the autosomal marker positions of the 1240K SNP set of the AADR dataset [23] in all of our simulations.

### The effect of random pseudo-haploidization (RPsH) on PCA calculations

Since the selected kinship methodology, PC-Relate, applies principal component analysis (PCA) to identify population structure, we first tested the effect of RPsH on PCA. We selected the British (GBR), Toscani (TSI), Iberian (IBS), and Finnish (FIN) populations from the 1000 Genome Project Phase 3 (1KG phase 3) dataset (404 samples) and randomized the diploid dataset with three different seeds. We performed smartpca analysis on the original diploid and the three random pseudo-haploidized dataset. According to our results, RPsH does not alter the PCA calculations significantly (Additional file 1: Figure S1).

### The effect of random pseudo-haploidization (RPsH) on kinship coefficient calculation

We assessed the effect of RPsH on kinship calculation by selecting 509 individuals from five populations with different population structure from the 1KG phase 3 dataset [25]. The five populations were as follows: FIN, GBR, TSI, Han (HAN), and Utah residents with Northern and Western European ancestry (CEU). In our experiments, we generated 100 different pseudo-haploid datasets from the original diploid data using different random seeds.

To study exclusively the effect of RPsH on kinship coefficient calculation, we included sample duplicates with different random pseudo-haploidization. This setup does not exclude differences between the genome structure of the test sample and the reference population, thus we selected random individuals from the GBR (HG00244.SG), FIN (HG00356.SG), CEU (NA12763.SG, NA12775.SG), and TSI (NA20798.SG) populations. This allowed us to overcome the interference of other effects, such as skewed recombination/segregation, differences in sequence alignment, genotyping, genome composition, or the population structure between the relatives. This idealized experimental setup is the equivalent of the monozygotic twin kinship relation, in forensics referred to as sample matching, while the maximal expected kinship coefficient (0.5) allows for the most sensitive analysis.

To study the effect of RPsH on true first/second-order relatives, we also selected samples with known family relations from the 1KG phase 3 dataset. (a) HG00702-HG00657 a parent-child relation from a Han population, where exactly 50% of genome is shared between the two samples, (b) NA20526-NA20792 siblings from a TSI population, where 50% of the genome comes from the same parents; however, a different subset of the markers are found in the sibs due to segregation and recombination, and (c) HG00124-HG00119 second-order relatives from a GBR population, where statistically 25% of genomes are shared. We calculated the kinship coefficient for each of the selected relatives (kin1/kin2) using their own reference population for the 100 different randomization and calculated the mean and the standard deviation of the estimated kinship coefficients. Knowing the expected kinship coefficients (0.5 for sample match, 0.25 for 1st-, 0.125 for 2nd-degree relations), we were able to validate that RPsH does not significantly alter the calculated kinship coefficient in these settings (Additional file 2: Table S1).

### The effect of overlapping marker fraction on the kinship coefficient calculation

The original PC-Relate algorithm was created to analyze modern fully genotyped diploid samples. However, in the case of ancient data, genome coverage and partial genotyping is one of the factors that has the greatest variability between samples. Kinship coefficient calculation is based on the IBD segments shared between two samples which can only be assessed at marker positions where both samples are genotyped. To investigate the effect of this factor, we defined a metric called overlapping marker fraction. We calculate this metric by dividing the number of markers where both samples are genotyped with the total number of markers in the dataset (1240K).

**Fig. 1:**
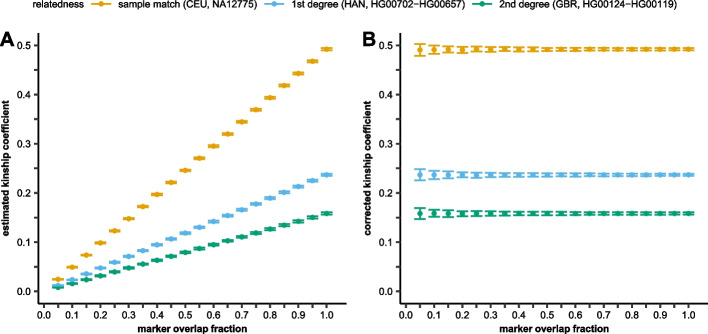
The mean and the 95% confidence interval of the **A** uncorrected and **B** corrected kinship coefficient between selected 1KG individuals (sample dup, known 1st and 2nd degree) at different marker overlap fractions

Using the 100 random pseudo-haploidized fully typed dataset of the previous experiment, we randomly depleted the markers between the selected sample dups and true 1KG relatives to a marker overlap fraction between 5 and 100% using different random seeds. We calculated the mean and SD of the estimated kinship coefficients between the selected sample pairs of the different randomizations for each overlap fraction (Additional file 2: Table S1). We visualized the mean and SD of the uncorrected kinship coefficients of a sample dup (CEU; NA12775.SG), a known first-degree relation (HAN, HG00702-HG00657) and a true second-degree relation (GBR, HG00119.SG-HG00124.SG) in Fig. 1A.

The results revealed that the kinship coefficient is a linear function of the marker overlap fraction. This allows a simple method for correcting the kinship coefficient value for low-coverage genomes by dividing the estimated kinship coefficient with the marker overlap fraction between the two samples. According to our simulation, the correction of estimated kinship coefficient for sparsely genotyped data resulted in reproducible kinship estimation regardless of the overlap fraction (Fig. 1B). As expected, low partially overlapping subset of markers would lead to less complete representation of the reference population and the test individuals thus regression of IBD/IBS based on the PC-Relate algorithm would have higher SD at low marker counts. Although the marker overlap fraction correction differs more than one magnitude between very low and high marker overlap fraction sample pairs, the analysis shows (Additional file 2: Table S1) that the correction itself does not introduce overall bias (the mean is statistically the same) and does not significantly multiply the error rate (4× increase in SD at 20× correction factor). Our results suggest that the increased SD of the method are likely due to the higher uncertainty of PCA.

### The effect of genotyping errors on the corrected kinship coefficient

**Fig. 2:**
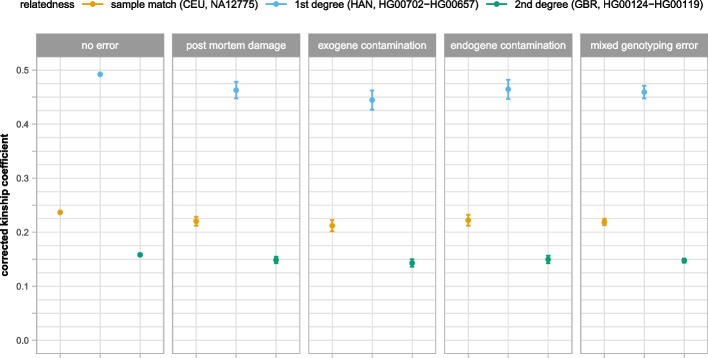
Effect of different aDNA-related genotype errors on the corrected kinship coefficient. In the last case (mixed error), we introduced an equal amount of post mortem damage, exogenous and endogenous contamination in the simulated data. The points represent the mean, and the error bars represent the 95% confidence interval of the corrected kinship coefficient between selected 1KG individuals (sample dup, known first and second degree)

We tested the effect of genotyping errors using the same dataset and sample dups/known first- and second-degree 1KG relatives as in the previous experiments. We simulated 4 scenarios: (1) only post mortem damage, (2) only endogenous contamination (using a random YRI individual as the contaminant), (3) only exogenous contamination, and (4) equal combination of the first three sources of genotype errors. In each scenario, we had approximately twice as many genotyping errors per individual introduced as in a typical aDNA dataset (see “Methods”). We calculated the mean and SD of the corrected kinship coefficients (Additional file 3: Table S2) between the selected samples. We visualized the mean and 95% confidence interval of the corrected kinship coefficients of the selected sample dup (CEU; NA12775.SG), true first-degree relation (HAN; HG00702-HG00657), and true second-degree relation (GBR; HG00119.SG-HG00124.SG) in Fig. 2.

In general, genotyping errors lower the mean and increase the SD of estimated kinship coefficient. The decrease in the corrected coefficients was proportional to the expected kinship coefficients. The largest effect (~9.6% lower kinship coefficient) was seen in case of the exogenous contamination. We speculate that it was likely due to the fact that in this scenario (although the genotyping error affects different subset of markers in different samples) the states of the markers were uniformly set to the homozygote major state leading to higher bias than that of random flip of minor/major state in different samples in the other scenarios. In our simulations even at the applied relatively high error rates (compared to experimental aDNA error rate), the largest effect was still significantly smaller than the 50% difference of expected kinship coefficients between different degrees of relations thus the estimated degree of relatedness for the analyzed relatives remained the same. However, we have to note that skewed IBD sharing and high genotyping error in the test individuals could lead to false (one-degree higher) classification of the analyzed relation.

### The effect of reference population selection on kinship analysis

**Fig. 3:**
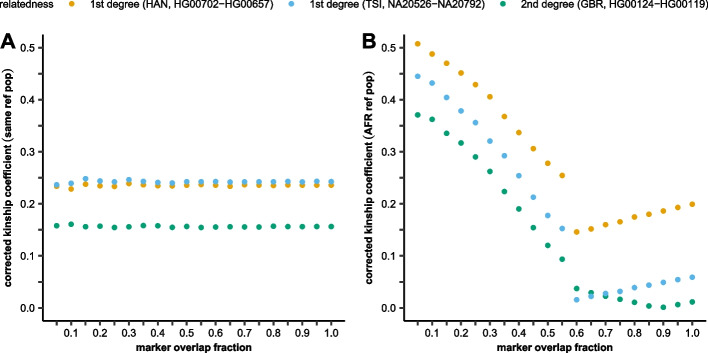
The effect of reference population choice and marker overlap fraction on the corrected kinship coefficients between known 1st–2nd-degree 1KG relatives. **A** The samples’ own populations used as references. **B** The AFR super-population was used for each sample

In this analysis, we wanted to investigate the scenario in which proper reference population is unknown or unavailable which is often the case for ancient samples. Using the same public 1KG phase 3 dataset and the three known relatives HG00702-HG00657 parent-child of Han population, NA20526-NA20792 siblings of TSI population, and HG00124-HG00119 second-order relatives of GBR population, we investigated the effect of the reference population on the calculated kinship coefficients. We tested three different scenarios: (1) the reference population was the same as that to which the selected individual belonged to; (2) the reference population was from a different super-population (AFR); (3) the reference population was from the same super-population as the selected individual (JPT for Han, IBS for TSI, FIN for GBR) (Fig. 3, Additional file 1: Figure S2). To study the effect of overlapping marker fractions in these more complex cases, the selected sample pairs were also marker depleted in the range of 100 to 5% overlap fractions.

The results revealed that a reference population with significantly different genetic background, like African for European samples, strongly corrupts the results. In this experimental setup, we lack a proper number of unrelated references; hence, IBS fractions are likely not represented and cannot be properly regressed out. Furthermore, when only a couple non-AFR individuals are included in the analysis depending on the marker overlap fraction, the EUR/EAS-specific markers are mostly excluded as the PCAngsd implementation uses a default 0.05 MAF marker pruning. Thus, at low marker overlap, mainly the AFR-specific markers are kept while at higher maker overlap slightly more EUR/EAS-specific markers are also included in the analysis. Based on the used marker set, the optimal number of eigenvectors and the underlying PCA-based regression of IBS components are expected to be different. We speculate that these differences could be the likely cause of the observed non-linear kinship coefficient estimates in Fig. 3B.

Using a super-population with similar genetic background to the sample gives very similar results as if its own reference population were used (Additional file 1: Figure S2).

### Effect of reference population selection on kinship analysis in a complex admixed family with multiple ethnic relations

To assess the choice of reference population in the kinship analysis of admixed individuals (often the case in ancient populations), we analyzed a complex admixed Cabo Verdean-Hungarian family with known pedigree. In this family, we had multiple old as well as recent admixes resulting in various admix component ratio individuals. WGS data was available for siblings (1st order), differently admixed half-sibs (2nd order), and 5th-order relatives as shown in Additional file 7: Figure S3.

**Fig. 4:**
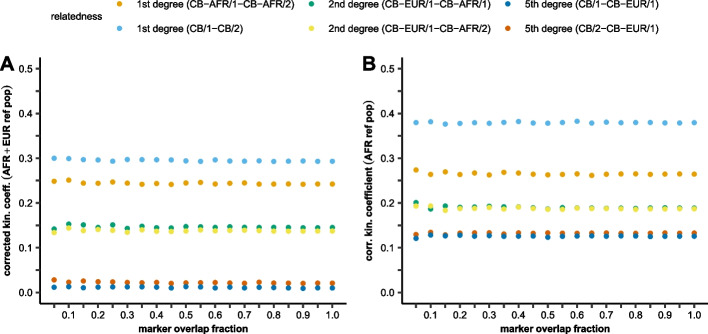
The effect of reference population choice and marker overlap fraction on the calculated kinship coefficient in a complex admixed modern family with 1st- (sibs), 2nd- (half-sibs), and 5th-degree relations, where individuals originated from populations with largely different genetic structure. Markers were depleted between the relatives to 5–100% overlap fractions. **A** The reference population was a combined set of AFR+EUR populations. **B** The reference populations were AFR populations

We tested two scenarios: the reference population was (1) only African (AFR) representing the majority of the admix sources in the tested samples; (2) both African and European (EUR) populations were included. Additionally, we performed marker depletion to investigate the effect of coverage in this complex scenario (Fig. 4).

The results in Fig. 4 demonstrate that in order to obtain realistic coefficient values in case of a complex admixture, a combined set of reference populations is required representing the population structure of all ancestors. Using just the majority source as reference significantly distorts the result.

To test whether random haploidization alters the kinship coefficient calculation compared to the better phased diploid data in this complex admixed case, we also performed this analysis from the original diploid dataset (Additional file 4: Table S3) with the AFR + EUR reference population. It was confirmed again that even in such complex admixed family, the differences due to RPsH were negligible.

### Statistical validation, assessment of technical errors

**Fig. 5:**
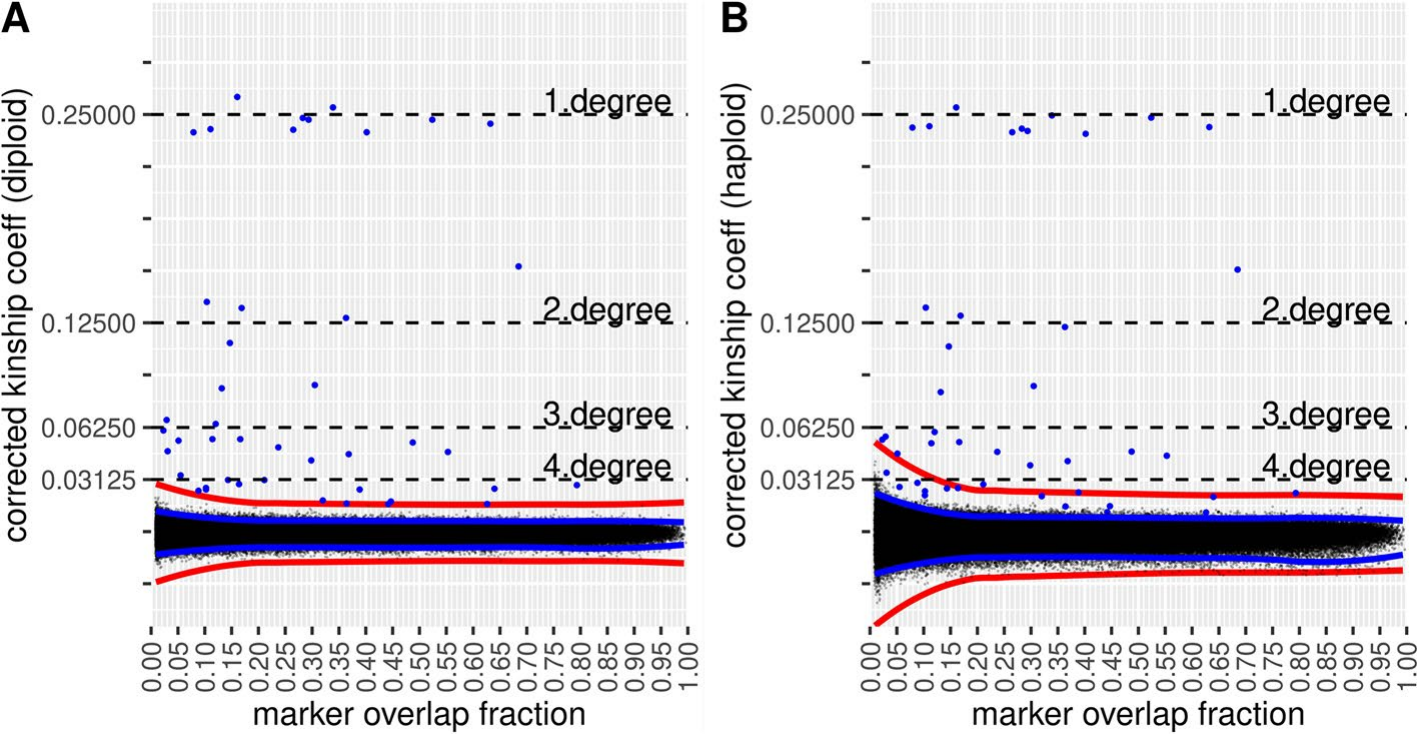
Corrected kinship coefficients calculated between 1020 EUR and EAS individuals from **A** diploid and **B** haploid dataset. In both cases, individuals were marker depleted between 100,000 and 1.2M markers to simulate partially typed data. The red line represents 6 sigma threshold from the mean. Individual kinship coefficients below the threshold are displayed as small black dots, while individuals above the threshold are displayed as larger blue dots. In case of haploid data, we marked all kinship coefficients that were above the 6 sigma threshold in the diploid dataset for better comparison. The blue lines show the 99% confidence interval of estimated kinship coefficients between unrelated individuals

We selected EUR and EAS individuals from 1KG phase 3 dataset (*n*=1020) and estimated the kinship coefficients between these individuals. Although there are a few true relatives in the selected individuals, the overwhelming majority of pairwise relations are expected to be unrelated, thus representing the variance of technical error of the whole analysis. To test the effect of overlapping genotyping fraction on the mean and standard error of the corrected kinship coefficient, we randomly depleted the marker set in these individuals between 100,000 markers and the fully typed marker count (~1.2M), amounting to 10–100% of the marker count of the original dataset. Using RPsH, we also created a pseudo-haploid dataset for comparison. We calculated the pairwise kinship coefficient matrix and corrected the estimated kinship coefficients by the marker overlap fraction. Using the pairwise matrix of 1020 individuals, we plotted the 519,690 kinship coefficients between all combinations of individuals for the diploid and haploid dataset (Fig. 5).

The variance of the corrected kinship coefficient depends on the marker overlap fraction between the test individuals (Fig. 5). Since the marker overlap fractions between any two ancient samples are different applying a pre-defined kinship coefficient threshold to identify relatives would lead to decreased sensitivity or specificity depending on the marker overlap fraction. In other words, the statistical power to differentiate relatives from unrelated depends on the marker overlap fraction and the same threshold should not be applied. However, based on the experimental variance of the corrected kinship coefficient observed in the analyzed dataset, the *Z* score (*N* standard deviation from the mean) could be used as a criteria to differentiate relatives from unrelated with the same sensitivity and specificity independent of the marker overlap fraction. Since in this experiment we could not exclude errors due to missing genome components from the reference populations, we used a conservative *N*=6 sigma threshold to identify biological differences. As expected, the haploid dataset resulted in higher variance due to random information loss especially at very low (<5%) marker overlap fractions. Although the marker overlap fraction correction differs two magnitudes (0.007–0.996) between very low and high marker overlap fraction sample pairs, the analysis shows that similar to the corrected kinship coefficient between relatives (Additional file 2: Table S1) the correction itself does not significantly multiply the error rate of unrelated samples. Most of the technical errors are expected to be the result of using very sparse data to regress out IBS to identify IBD fragments by PC-Relate. In spite of the higher variance, the estimated kinship coefficients show very high correlation between the fully typed (~1.2M high-quality diploid markers) diploid and the corrected coefficients of the partially typed diploid and haploid datasets (*R*=0.9998 and *R*=0.9993 respectively) compared to the correlation with the uncorrected estimates (*R*=0.749 and *R*=0.751; Additional file 5: Table S4). Our result suggests that the applied pseudo-haploidization and correction in the marker depleted experimental data does not introduce overall bias. Our method identified all known 1st- and 2nd-degree relatives that were included in the analyzed subset of 1KG EUR/EAS individuals and indicated a couple of additional distant 3rd or 4th relatives (Additional file 5: Table S4). Our analysis shows that 4th-degree relatives are expected to be above the 6 sigma threshold in the diploid data except at very low overlapping marker fractions (<2% ~ 17,000 overlapping markers). In case of haploid data, establishment of 3rd-degree relatedness is possible even from low marker overlap fractions, and establishment of fourth-degree relatedness is possible when the sample pair has >10% marker overlap fraction (equal to roughly ~85,000 markers). However, in case of 4th-degree relatives depending on the overlapping genotyping fraction, the estimated confidence interval of corrected kinship coefficient, and true biological variation, it is expected to have more false positive/negative and uncertain kinship estimations at low marker overlap fractions.

### Kinship analysis of ancient samples with known relations using kinship coefficient correction

To show that our methodology is also suitable for ancient data, we analyzed low-coverage ancient sequences with known family relations. In the first example, we present the analysis of a known father-son (first degree) relation of two Medieval samples. From both remains, we had two types of biological samples, bone powder taken from the teeth and from the *pars petrosa*. From the father, we had three parallel DNA isolates and NGS libraries, two prepared from *pars petrosa* and an additional one from teeth. From the offspring, we had one DNA isolate and NGS library prepared from both types of biological samples. Altogether, we had 3+2 NGS sequences with largely different genome coverages (0.87×–11.9×) from these two ancient individuals. Accordingly, we assessed the robustness of our correction method on the 6 combinations of these datasets. We present the uncorrected and corrected kinship coefficients calculated from this data in Table 1.

**Table 1:** Correction of kinship coefficient for different genome coverage of ancient samples with known first-degree relation. High, medium, and low refer to coverage levels

**Sample 1**	**Sample 2**	**Marker overlap fraction**	**Relation**	**Expected kinship coeff**	**Uncorrected kinship coeff**	**Corrected kinship coeff**
Father high	Child high	0.933894	First degree	0.25	0.22883	0.24502
Father high	Child low	0.377769	First degree	0.25	0.09179	0.24297
Father medium	Child high	0.730011	First degree	0.25	0.17604	0.24114
Father medium	Child low	0.296127	First degree	0.25	0.07150	0.24144
Father low	Child high	0.511753	First degree	0.25	0.12239	0.23915
Father low	Child low	0.207545	First degree	0.25	0.05014	0.24160
Father high	Father medium	0.778247	Sample matching	0.5	0.38284	0.49193
Father high	Father low	0.545866	Sample matching	0.5	0.27020	0.49499
Father medium	Father low	0.427389	Sample matching	0.5	0.20863	0.48814
Child high	Child low	0.354511	Sample matching	0.5	0.17286	0.48761
**Sample**	**Sample type**	**Coverage**	**Typed marker count (1240k)**			
Father high	Teeth	11.997	1,148,973			
Father mid	Petrosa	3.057	896,623			
Father low	Petrosa	1.546	630,405			
Child high	Petrosa	5.510	1,075,845			
Child low	Petrosa	0.879	435,005			

In the second example, we reanalyzed a published group of five related males from the Corded Ware Culture (2500–2050 BCE) with first-, second-, third-, and fourth-degree kinship relations [19]. In Table 2, we present the family relations with uncorrected and corrected kinship coefficients calculated by our methodology from public ancient 1240k data.

**Table 2:** Kinship analysis of a large ancient Corded Ware family with multiple 1st- to 4th-degree of relations

**Sample 1**	**Sample 2**	**Marker overlap fraction**	**Relation**	**Expected kinship coeff**	**Uncorrected kinship coeff**	**Corrected kinship coeff**
I1538	I1541	0.039828	First degree	0.25	0.01036	0.26006
I1540	I1541	0.079863	First degree	0.25	0.01959	0.24534
I1534	I1541	0.047882	Second degree	0.125	0.00715	0.14938
I1538	I1534	0.025735	Second degree	0.125	0.00335	0.13003
I1538	I1540	0.042478	Second degree	0.125	0.00456	0.10730
I1541	I0104	0.216185	Second degree	0.125	0.03394	0.15700
I1534	I1540	0.049813	Third degree	0.0625	0.00405	0.08123
I1538	I0104	0.107634	Third degree	0.0625	0.00846	0.07864
I1540	I0104	0.22405	Third degree	0.0625	0.01812	0.08088
I1534	I0104	0.129456	Fourth degree	0.03125	0.00645	0.04982
**Sample ID**	**Coverage**	**Typed marker count (1240k)**				
I0104	4.184	962767				
I1534	0.158	164095				
I1538	0.126	135269				
I1540	0.298	285866				
I1541	0.294	276299				

These individuals were analyzed in the original READ manuscript [26] where relations were identified up to the 2nd degree except the one between I1538 and I1540, and all of the 3rd and 4th relations were inferred from only the family relations.

### Kinship analysis of ancient samples from the AADR 1240K dataset

We performed kinship analysis on 2136 ancient Eurasian individuals from the AADR 1240K dataset that had more than 100K genotyped markers. Since no manual curation or additional matching reference population was used, we filtered potential relatives above 0.046875 corrected kinship coefficient (~3rd–4th degree of kinship). Our analysis identified 410 related individuals in 184 kin groups (Additional file 6: Table S5). All sample duplicates (*N*=26), and joint datasets (*N*=30) of the same sample were identified. Curiously, we identified sample duplicates with different master IDs in the AADR dataset published in different manuscripts (I1526-NEO232; I7782-NEO298; I8295-NEO230; I8296-NEO231) where all four sample pairs were from the same geological site and belonged to the same population and haploid typing was identical or nearly identical as some branch defining markers were likely missing due to coverage differences. We also identified a likely sample mix [27] where two individuals (MJ-15 Ukraine_IA_WesternScythian.SG and MJ-35 Ukraine_Cimmerians_o2.SG) had 0.5 corrected kinship coefficient equivalent with sample match (or monozygotic twin) but had different population assignment. All of these individuals had same sex and identical/nearly identical mitochondrial and Y haplogroups as well. Furthermore, we identified all of the 111 previously identified kinship relations from the AADR dataset. Three uncertain (1st or 2nd) relatives indicated in the AADR dataset (I8502, I8524; MK5001, MK5004 and KBD001, KBD002) could be classified as 2nd-degree relatives by our analysis. We reclassified three kin pairs indicated as 1st-degree relatives as 2nd-degree relatives (RISE1163, RISE1169; RISE1168, RISE1173 and RISE1168, RISE1169). In addition to the published data, within the 184 kin groups our approach indicated 6 new 1st-degree, 108 2nd-degree, 144 3rd-degree, and 40 4th-degree relations between a total of 279 new relatives (Additional file 6: Table S5). In a few cases, when an appropriate reference population was not present in the dataset, it is not possible to establish appropriate kinship relations as the IBS of minor genetic components cannot be regressed out. Consequently, in those cases, the correction resulted in invalid distant 3rd–4th-degree kinship relations highlighted with red in Additional file 6: Table S5.

To test the sensitivity of our analysis, we used READ [26] to validate our findings. As READ depends on the proper reference population and uses a global threshold to distinguish between unrelated and potential kins, the join set of 2136 individuals cannot be analyzed together. We selected the top 10 populations with the highest number of individuals and performed the READ analysis separately. READ identified relatives up to the 2nd degree. In the selected populations, READ identified no additional relatives compared to our methodology. For each identified relative, the degree of kinship was matching between the two methods. In this comparison, our method indicated one additional 2nd-degree relation (AITI_95_d and AITI_98) that was missed by READ. In this case, the samples have very low genome coverage (0.145× and 0.402×) and only ~0.05% marker overlap fraction. While the corrected kinship coefficient was significantly above the 3rd-degree (0.0625) relation, it was less (0.1004) than the expected 0.125 corresponding to 2nd degree suggesting that these relatives share less than expected genome portions due to true biological variation (Additional file 7: Table S6). We had very similar scenario in case of the missed 2nd-degree relation between the I1538 and I1540 CWC individuals (Table 2) suggesting that READ is less sensitive when the marker overlap is low and the shared genome fraction differs significantly from the statistically expected mean.

## Discussion

Identification of relatives from the genomic data of ancestors is of great interest as it allows the study of family relationships, but it is also a precondition for most population genetic analyses to exclude close relatives from datasets (e.g., ADMIXTURE, PCA). To date, the best analysis tools were able to indicate mainly first- and second-degree relatedness from very low-coverage ancient samples [26, 28–30]. Based on simulated data, lcMLkin can accurately infer kinship up to the 3rd degree from 2× genome coverage when the F_ST_ is low between the reference population and analyzed data [28]. However, the majority of aDNA data is below 2× genome coverage. In these data, most markers are represented by one read/genotype only. It is untested whether it is possible to infer comparable diploid genotype likelihoods suitable for lcMLkin from very low-coverage data. The recent heuristic method READ (Relationship Estimation from Ancient DNA) infers relatedness up to 2nd degree from as low as 0.1× coverage sequence data [26]. In the most comprehensive AADR ancient genome data set [23], the majority of the indicated kinship relations are 1st degree and the handful of indicated 2nd-degree relations in all cases are uncertain. These samples are labeled with 1d.or.2d.rel tag.

Diploid variant calling and genotype likelihood-based methods with the extra information of rare alleles allow better phasing and identification of IBD fragments leading to improved kinship coefficient estimations from deeply genotyped WGS data. Accordingly, some methods attempt to infer genotype likelihoods or diploid genotype calls from low-mid genome coverage (2–4×) data [8, 28, 31]. KING, a method that was developed to be used for fast and robust kinship coefficient estimation from low amounts of fully typed diploid markers (5–150k), can infer up to 3rd-degree relations from approximately 150k markers or 1st–2nd-degree relation from even as low as 5k diploid markers [8]. Even though these tools are used to analyze low marker count ancient samples, the assumption implicit in these methods that the data is sufficiently high-quality diploid is often false in case of low marker count extremely low-coverage ancient samples. Accordingly, when comparing samples of different genome coverage, the inferred genotype likelihoods or diploid variants from low/variable genome coverage samples could lead to major bias.

To overcome these difficulties and mitigate the main genotyping biases in case of low-coverage ancient samples, we used a combination of strategies to account for the effects caused by PMD and varying low genome coverage. We used random allele sampling that is the gold standard methodology when performing PCA and other population genetic analyses on ancient samples, as it leads to statistically equal genotype likelihoods of genotyped markers regardless of the genome coverage. To avoid excessive, variable amounts of false positive variants due to the variable rate of PMD, exogenous DNA contamination, and technical errors (alignment artifacts), we restricted our analysis to the already known biallelic, high-frequency, and population-informative SNPs of the 1240K AADR dataset. This strategy perfectly aligned with our choice of kinship analysis method since the PC-Relate algorithm uses PCA to differentiate between IBD/IBS fragments.

We have demonstrated that random pseudo-haploidization of data in our analysis pipeline does not affect the result of kinship analysis (Additional file 2: Table S1). This is also confirmed by the PCA analysis, showing that the same modern individual from diploid or different pseudo-haploidized data had nearly identical PCA components (Additional file 7: Figure S1).

Overlapping marker fraction, according to our study, is the major factor influencing the calculated kinship coefficient of partially genotyped samples in our analysis pipeline. Our simulations revealed that the overlapping marker fraction and the calculated kinship coefficient had a strong linear correlation (Fig. 1, Additional file 2: Table S1). Although the PC-Relate algorithm does not require the specification of the underlying population structure of the analyzed relatives, we have shown that a proper reference set is required for the analysis. As expected, the samples’ own reference population resulted in proper kinship coefficients, but using reference from a different super-population corrupted the results (Fig. 3). On the other hand, using the samples’ super-population as reference resulted in comparable although slightly higher kinship coefficients compared to the proper reference population (Additional file 7: Figure S2) proving the robustness of the PC-Relate algorithm. This reference bias is not amplified by the applied correction for marker overlap (Fig. 4); however, it could lead to the false identification of distant relatives. We also tested the effect of reference population choice in a complex Creole/European admixed Cabo Verdean-Hungarian family with known 1st- to 5th-degree family relations. We have shown that the best result is achieved when all super-populations of the sources are included in the reference population set (Fig. 4). Comparing the analyses of pseudo-haploid and diploid data for this complex admixed family confirmed the robustness of our approach, as we got nearly identical results (Additional file 4: Table S3).

In the statistical evaluation using the downsampled modern diploid/pseudo-haploid data, we simulated marker counts similar to aDNA data. We applied the same minimum 100,000 genotyped markers per individual threshold that was used in the analysis of 2136 selected ancient individuals from the AADR dataset. This equals roughly 0.08× genome coverage considering the ~1.15M autosomal markers of the 1240K marker set. Thus, the simulated data had similar marker counts and distribution as the analyzed AADR dataset. Accordingly, pairwise marker overlap was <5% (<57,000 markers) between 3.72 and 3.46% of the analyzed sample pairs (ancient and modern respectively). Our analysis shows that when proper reference population is available, the applied method is suitable to identify relations up to the 4th degree from low to high coverage mixed samples (Fig. 5).

We also confirmed the robustness of our methodology on real ancient data with known family relations. Our analysis showed that in the case of a medieval Hungarian family, a general modern European reference super-population gave appropriate results. Despite the fact that the uncorrected kinship coefficients varied highly due to the different genome coverages, our methodology resulted in reproducible corrected kinship coefficients consistent with the known family relation in each case (Table 1). In the second example, we reanalyzed published kinship relations from Corded Ware Culture samples [26]. Compared to the READ software which could indicate relations up to the second degree of kinship and even missed one second-degree relation, our approach could properly identify all relations up to 4th degree from this large ancient family with very low/variable genome coverages (0.12×–4.18×), underlining the efficiency and usefulness of our approach (Table 2).

Our results exposed both the advantages and the limitations of our method. Although RPsH combined with the choice of the 1240K marker set in our study allowed us to overcome genotyping bias of low-coverage ancient samples, it clearly restricts the analysis to populations that are properly represented by these markers. In the PC-Relate algorithm, PCA is used to regress out the population-specific IBS components. Using linear regression to fit individuals to the model, all the remaining non-regressed PC components are calculated as IBD. Thus, insufficient amount of reference individuals, improper or missing population components in the reference, or marker sets that are lacking informative markers of the tests lead to underestimation of IBS and inflated kinship coefficient estimation. The greater the difference between the structure of related individuals and the reference populations, the greater fraction of IBS is accounted incorrectly as IBD which can seriously bias small kinship coefficients representing very distant kinship relations. Accordingly, the current 1240K marker set is less suitable for the analysis of extremely old samples, and for small isolated populations, because these supposedly have less informative markers in this marker set, and also have insufficient reference populations in the current genome databases. Furthermore, while PC-Relate kinship coefficient estimator is known to be appropriate even in inbreed populations [24], we have to caution that in case of inbreed or small drifting populations extra care has to be taken to confirm that the test individuals are analyzed with their own reference population. When no prior knowledge exists on the reference population, F_ST_ or FastNGSAdmix [32] analysis could be used as an objective method to select individuals best matching our test individual’s genome structure as a reference population.

Genotyping error simulations show that approximately double error rate compared to typical experimental aDNA data leads to 5.4–10.4% proportionally lower corrected kinship coefficient than the expected kinship coefficient in our workflow (Fig. 2, Additional file 3: Table S2). The mean corrected kinship coefficient of the validated sample dups and 1st relatives of the experimental 2136 AADR individual was 0.48 and 0.24 respectively (approximately ~4% lower from the expected). This is in accordance with the mean X contamination rate (1.28%) of ancient individuals of the AADR V42.2 dataset suggesting that our kinship-estimation method can be safely used on typical aDNA data. Nevertheless, analysis of highly contaminated (CRITICAL/FAIL) samples containing higher rate of genotyping errors (>5%) could lead to underestimation of corrected kinship coefficient and as a result to underestimation of the degree of relation especially in case when the relatives share less than the expected IBD fragments due to true biological variation.

The unsupervised analysis of 2136 ancient individuals of the 1240K AADR dataset (Additional file 6: Table S5) demonstrated that our method can identify real 1st–4th degree of relatedness from very low-coverage ancient damaged samples and fails only when the proper reference population is not present in the dataset. Comparison with READ showed that our method has better sensitivity, offers improved performance, and scales better on multi-core machines (Additional file 7: Table S6). On the other hand, our results show that the 1240K marker set was sufficient to properly analyze 4000-year -old ancient Corded Ware Culture individuals with a modern Eurasian reference population, suggesting that the majority of the high-frequency EUR informative markers were already present at this age.

According to our results, the used method had slight downward (2–4%) bias in the analyzed 1KG dups and first-degree relatives and also in the validated first-degree ancient samples. However, this downward bias is also present in the kinship coefficient estimation of fully typed diploid 1KG relatives suggesting that the original PC-Relate algorithm and not the applied correction or pseudo-haploidization is accountable for this bias. This is also supported by our simulations on the corrected kinship coefficient calculated from marker depleted pseudo-haploid data and the original fully typed diploid data (Additional file 2: Table S1, Fig. 1B) and the very high correlation between the kinship coefficient calculation of marker depleted pseudo-haploidized and the original fully typed 1KG data (Additional file 5: Table S4). On the other hand, the mean of the corrected kinship coefficient of the indicated 2nd-degree relatives (*n*=119) of experimental AADR data is 0.1274 (Additional file 6: Table S5) that is a slightly over the expected value (~2% relative difference) suggesting that the bias could originate from more than a single factor.

Our analysis revealed new possibilities to improve kinship analysis from low-coverage ancient data. Diploid typing with pre-capture enrichment could result in higher sensitivity even at lower marker overlap fraction as seen in Fig. 5. However, this is only feasible when a sufficient number of individuals are available from the matching reference populations. According to our analysis, ~50–100 unrelated individuals are sufficient as a reference in case of modern samples. We speculate that in case of populations with less complex genome structures (like pre-iron age populations), a smaller number of unrelated individuals could likely represent the population structure properly. This is also demonstrated in the case of the validated relations of the analyzed CWC individuals where the analysis resulted comparable kinship coefficient estimates using modern EUR individuals as a reference or the 25 Czech, Latvian, Estonian, and German CWC individuals of the AADR dataset (Table 2, Additional file 6: Table S5). Alternatively, using larger marker sets would increase the number of overlapping markers between individuals resulting in higher sensitivity from the already available low-coverage WGS data. The increasing number of aDNA studies should identify proper reference populations and suitable high-frequency marker sets for cases that are difficult to analyze at present.

**Fig. 6:**
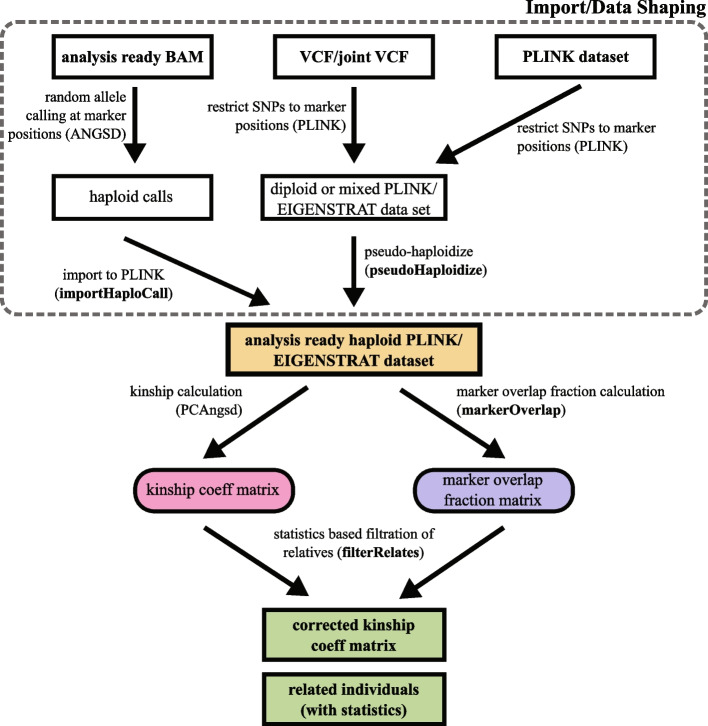
Step-by-step workflow to analyze kinship relation of low-coverage ancient/modern samples from various data sources. Additional tools presented in this manuscript are denoted with bold letters

To facilitate the evaluation and use of our approach, we provide a practical workflow (Fig. 6, Additional file 8: Note S1) for kinship analysis of low-coverage genome data.

Our workflow is based on publicly available free software ANGSD, PLINK, and PCAngsd. Additionally, we also provide the correctKin tool [https://github.com/zmaroti/correctKin] to import and shape data, calculate the pairwise overlapping marker fraction, and filter relatives based on the empirical error model.

## Conclusions

In summary, our proposed methodology is capable of reliably identifying the relatedness up to the 4th degree from low-coverage genome data, redefining the limits of kinship analysis from low-coverage ancient or badly degraded forensic WGS data.

## Methods

### Used software and datasets

The software requirements and the detailed instructions to perform the analysis workflow from various data sources are described in Additional file 8: Note S1.

In all of our analysis, we used the genome coordinates of 1240K SNP set from Allen Ancient DNA Resource (AADR) [23]. For marker overlap simulations, we used two different full-typed modern datasets: the 1000 Genomes Project Phase 3 data [25], and a large admixed Cabo Verdean-Hungarian family of known pedigree with first- (siblings), second- (half siblings), and fifth-degree relatives from our anonymized clinical biobank. The variants of the joint VCF (Variant Call Format) files were filtered for the 1240K SNP coordinates and imported into plink 1.9 binary format [33, 34].

To test the effect of genome coverage on the estimated kinship coefficients from real ancient data, we used our unpublished 1240K genotype data of a known medieval parent offspring. The dataset contains low-coverage partially typed pseudo-haploid genotype data from 3+2 separate library preparations from different biological samples of the analyzed individuals. We deposited the unpublished medieval datasets in PLINK 1240K binary format presented in this manuscript at Zenodo [34].

The public AADR V42.4 1240K dataset [23] was used to validate our methodology on a wide variety of ancient individuals. We included only ancient samples with more than 100K genotyped markers (*N*=2810). We excluded samples older than 8000BC (*N*=216) as older samples were very few and were lacking proper number of samples as a reference population (Additional file 7: Figure S4). To avoid analyzing individuals with very few and/or inappropriate reference populations, we restricted the analyzed samples by their geo location (in between the Longitude -12 – 120 and Latitude 28 – 65) excluding 458 individuals (Additional file 7: Figure S5). After filtration, the resulting dataset contained 2136 ancient individuals (Additional file 6: Table S5).

### New bioinformatics tools

To aid easy importing, manipulating, and analyzing the genotype data in our proposed workflow, we created the essential tools:

- *importHaploCall*to import pseudo-haploid genotype calls from the ANGSD
- *pseudoHaplo*to perform RPsH using a diploid dataset
- *markerOverlap*to calculate the pairwise marker overlap fraction matrix
- *filterRelates*to correct kinship coefficient, and filter relatives based on error model and/or hard kinship coefficient threshold

To study the effect of partially genotyped markers in a controlled fashion and comparing results with the analysis of the fully genotyped modern samples we used

- depleteMarkersto simulate the desired marker overlap fraction between selected samples
- depleteIndivsto simulate a random partially genotyped sample cohort

The tools work with the main genotype data formats (PLINK, EIGENSTRAT, PACKEDANCESTRYMAP). We documented the usage and options of the new tools with command examples in Additional file 8: Note S1. Tools are available in zenodo and the GitHub repository (https://github.com/zmaroti/correctKin) [35, 36].

### Random pseudo-haploidization and pairwise overlapping marker fraction calculation

We defined the overlapping marker fraction between two samples as the number of markers typed in both samples divided by the number of all markers in the dataset.

Using our “*pseudoHaplo*” tool, we created 100 randomly pseudo-haploidized datasets from the fully typed modern diploid dataset using different random seeds. In all of the presented examples, we used our own tool “*markerOverlap*” to calculate the pairwise overlapping marker fraction matrix of samples used for the kinship coefficient correction [35, 36].

### Principal component analysis

We selected the GBR, TSI, IBS, and FIN populations from 1KG dataset (404 samples) and randomized the diploid dataset with three different seeds. We performed smartpca [37, 38] analysis on the original diploid and the three random pseudo-haploidized dataset with the “*inbreed: YES*” option. We used the R (version 4.0.5) [39] and the ggplot2 R package (3.3.5) [40] to visualize the individuals on the PC1 and PC2 axes (Additional file 7: Figure S1).

### Simulating the effect of low coverage from fully typed modern datasets

To study the effect of coverage and the resulting lower genotyping percentage on the kinship coefficient calculation in a controlled fashion, we used “*depleteMarkers*” to randomly deplete markers from a fully typed (PLINK, EIGENSTRAT) dataset, resulting in the desired percentage of marker overlap between two samples [35, 36]. Using this tool, we simulated the overlapping marker fraction in the selected samples in the range of 5–100% with step of 5 percentages.

To assess the technical error of low/variable coverage data on the whole workflow, we selected 1020 fully typed diploid Eurasian samples (CEU, IBS, GBR, FIN, TSI, CDX, CHB, CHS, JPT, KHV populations) of the 1KG phase 3 dataset. We applied “*depleteIndivs*” to create a random, partially typed sample cohort with marker count between 100,000 and the full 1,150,639 markers. From the partially typed diploid dataset, we also created a pseudo-haploidized dataset using the “*pseudoHaplo*” tool [35, 36]. We performed kinship analysis with PCAngsd and corrected the estimated kinship coefficients according to the marker overlap fraction of sample pairs on the partially genotyped datasets. We compared the results with the estimated kinship coefficients using the original fully typed diploid dataset.

### Simulation of aDNA-related genotyping errors

PLINK and EIGENSTRAT data format were designed for biallelic markers. There are only 4 possible allelic states (homozygote major allele, homozygote minor allele, heterozygote major/minor, and missing), thus any other nucleotide that is different than the minor or major allele cannot be represented and the allelic state of samples with invalid alleles are set to the “missing” state at such marker positions.

Based on the data format restriction, the three typical aDNA-related genotype errors can be simulated in the following ways for pseudo-haploid PLINK dataset:

- Post mortem damage; if the C->T or G->A conversion leads to different nucleotide than the minor or major allele, the state is set to “missing,” otherwise, if the minor and major alleles are C/T, T/C, G/A or A/G, the homozygote minor and major states are flipped.
- Exogenous (non-human DNA) contamination; since the exogenous DNA consist of mainly DNA of microorganisms (usually in ancestral state), it leads to excessive homozygote major allele, thus random subsets of markers are set to the homozygote major allele state.
- Endogenous (human DNA) contamination; random subsets of markers are set to the state of the same markers genotyped from another sample (theoretically, the largest number of SNPs are expected to be flipped in case the population has the largest FST from the test individuals or practically if the test is contaminated with sample from a very different population).

From most population genetic analyses, highly contaminated samples are excluded. In the comprehensive AADR ancient dataset, the following criteria is used to mark bad-quality sequences:

- ANGSD X contamination (applicable only for males) 0.02–0.05="QUESTIONABLE", >0.05="QUESTIONABLE_CRITICAL" or "FAIL”.
- mtcontam <0.8 is "QUESTIONABLE_CRITICAL", 0.8-0.95 is "QUESTIONABLE", and 0.95–0.98 is recorded but "PASS", gets overridden by ANGSD X contamination.

Accordingly, the 1240K v42.2 AADR dataset (*n*=3589 ancient samples) 157 is marked CRITICAL/FAIL (>5% error rate), while the mean of the X contamination rate of all ancient samples is 1.28%.

We simulated the three different errors separately and also made a mixed case where all three error types were introduced in equal amount leading to the same total error rate. In all cases, we had maximum total genotyping error rate of 5% (the threshold of CRITICAL/FAIL tag of the AADR criteria). Accordingly, each sample had random 0–5% genotyping error, leading to an overall ~2.5% genotype error rate of the whole dataset that is roughly the double of the genotyping error rate of the experimental AADR aDNA dataset. In each simulation, we used 100 different randomizations with different random seed and calculated the mean and SD of the corrected kinship coefficients.

### Uncorrected kinship coefficient estimation

Kinship coefficient estimation was performed by the PCAngsd [41] software (version 0.99) from the ANGSD package [7] that implements a fast parallelized kinship calculation from PLINK or EIGENSTRAT format based on the PC-Relate algorithm [24] with the “*-inbreed 1 -kinship”* parameters.

## Supplementary Information

**Additional file 1: Figure S1.** Comparison of PCA using diploid and pseudo-haploid data. **Figure S2.** Effect of reference population (same super-population) on kinship coefficient. **Figure S3.** Pedigree of complex admixed modern family. **Figure S4.** The date distribution of ancient AADR individuals. **Figure S5.** Geographical distribution of ancient AADR individuals.

**Additional file 2: Table S1.** Effect of RPsH and marker overlap on kinship coefficient.

**Additional file 3: Table S2.** Effect of genotyping errors on kinship coefficient.

**Additional file 4: Table S3.** Comparison of kinship analysis of a complex admixed family using pseudo-haploid and diploid data.

**Additional file 5: Table S4.** Comparison of kinship coefficient correction using experimental 1KG Phase III data.

**Additional file 6: Table S5.** correctKin analysis of 2126 ancient AADR individuals.

**Additional file 7: Table S6.** Comparison of correctKin with READ software.

**Additional file 8: Note S1.** Installation requirements and a step-by-step method description including detailed usage examples to perform correctKin analysis from various data sources.

**Additional file 9.** Review history.

## Data Availability

The extra tools described and used in this manuscript were deposited to Zenodo [35]. Our software with potential future updates is also available in the GitHub repository (https://github.com/zmaroti/correctKin) [36]. The raw data of the medieval dataset can be accessed at European Nucleotide Archive (http://www.ebi.ac.uk/ena) under the accession number PRJEB47418 [42]. We deposited the medieval dataset and the subset of 1KG Phase III individuals presented in this manuscript at in PLINK 1240K binary format [34]. The complex admixed modern family data that supports the finding of this study are not publicly available due to them containing information that could compromise research participant privacy/consent. The anonymized 1240K AADR PLINK subset of this data without phenotype information is available on request from the corresponding author (ZM). Access to the data requires noncommercial, research only usage approved by your ethical committee.
