# Supplementary material for: correctKin: an optimized method to infer relatedness up to the 4th degree from low-coverage ancient human genomes
Source: Genome Biol. 2023 Feb 28;24:38. doi: 10.1186/s13059-023-02882-4 (PMC9972692; doi:10.1186/s13059-023-02882-4)
Supplement: Supplementary file 1 — Additional file 1: Figure S1. Comparison of PCA using diploid and pseudo-haploid data. Figure S2. Effect of reference population (same super-population) on kinship coefficient. Figure S3. Pedigree of complex admixed modern family. Figure S4. The date distribution of ancient AADR individuals. Figure S5. Geographical distribution of ancient AADR individuals. [file 13059_2023_2882_MOESM1_ESM.docx]

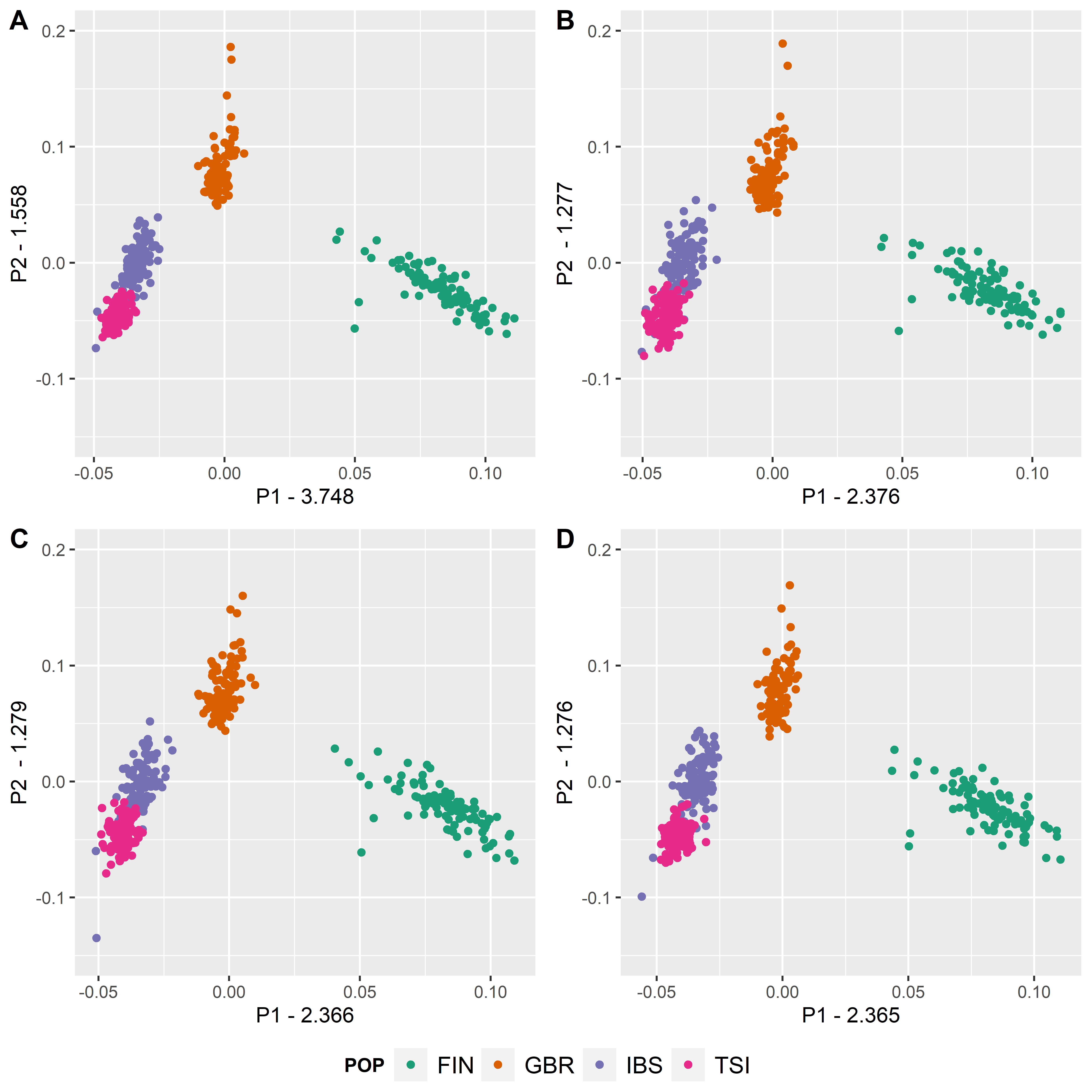


**Figure S1.** Random pseudo-haploidization (RPsH) of diploid data does not significantly alter PCA analysis. Individuals from the TSI, GBR, FIN and IBS populations were used from the 1KG phase 3 data set. A) PCA from the original diploid data set B-D) RPsH data set with three different random seeds.


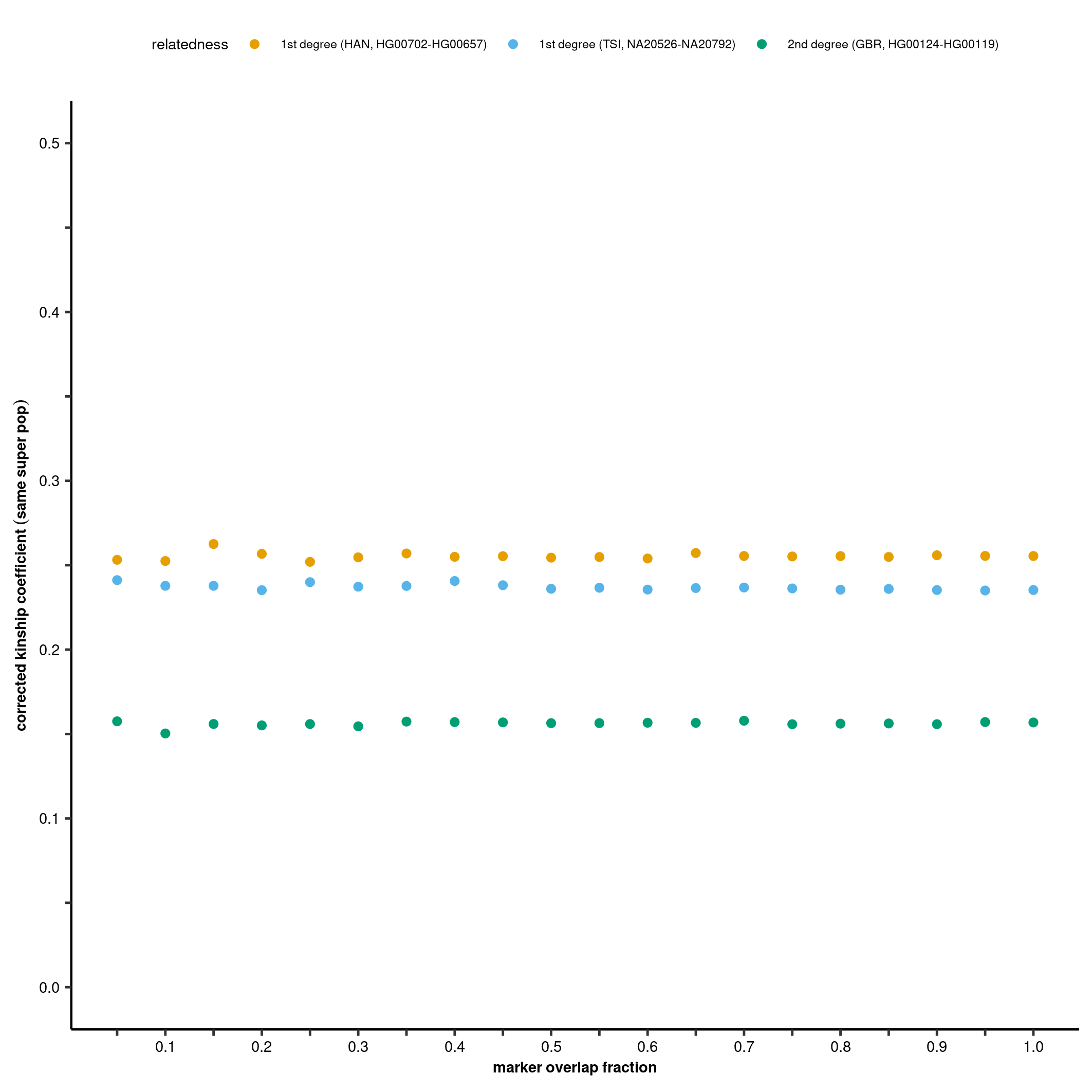


**Figure S2.** The effect of reference population choice and marker overlap fraction on the calculated kinship coefficients between selected 1^st^ -2^nd^ degree relatives. Markers were depleted between the relatives to 5-100% overlap fractions. The reference population was from the same super-population (JPT, IBS and FIN) as the selected individual (HAN, TSI and GBR) was derived from. The calculated kinship coefficients of fully typed data agrees with the expected coefficients and nearly identical with the values that were calculated using sample matched reference populations (HAN, TSI, GBR respectively).


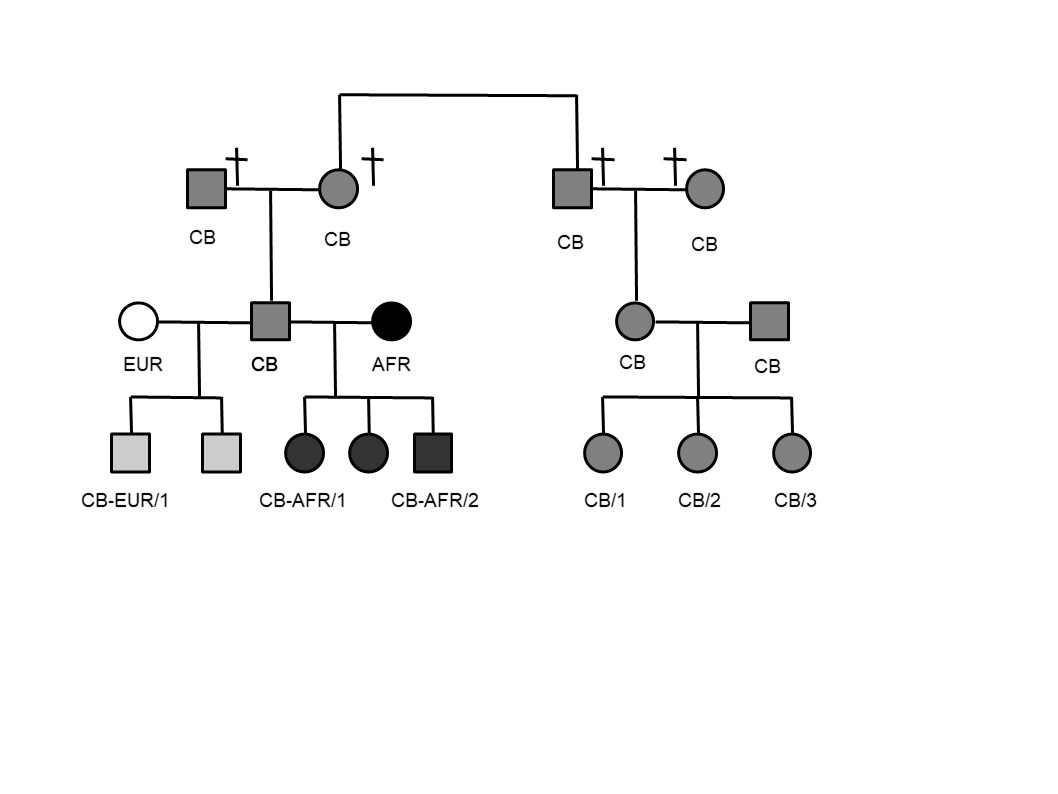


**Figure S3.** Pedigree of a complex admixed modern family with Cabo-Verdean (CB, ~50%-50% old EUR/AFR admix), AFR (100% African), EUR (100% Hungarian) family members and recently admixed offsprings. WGS data was only available from third generation individuals denoted with numbers in their ID.


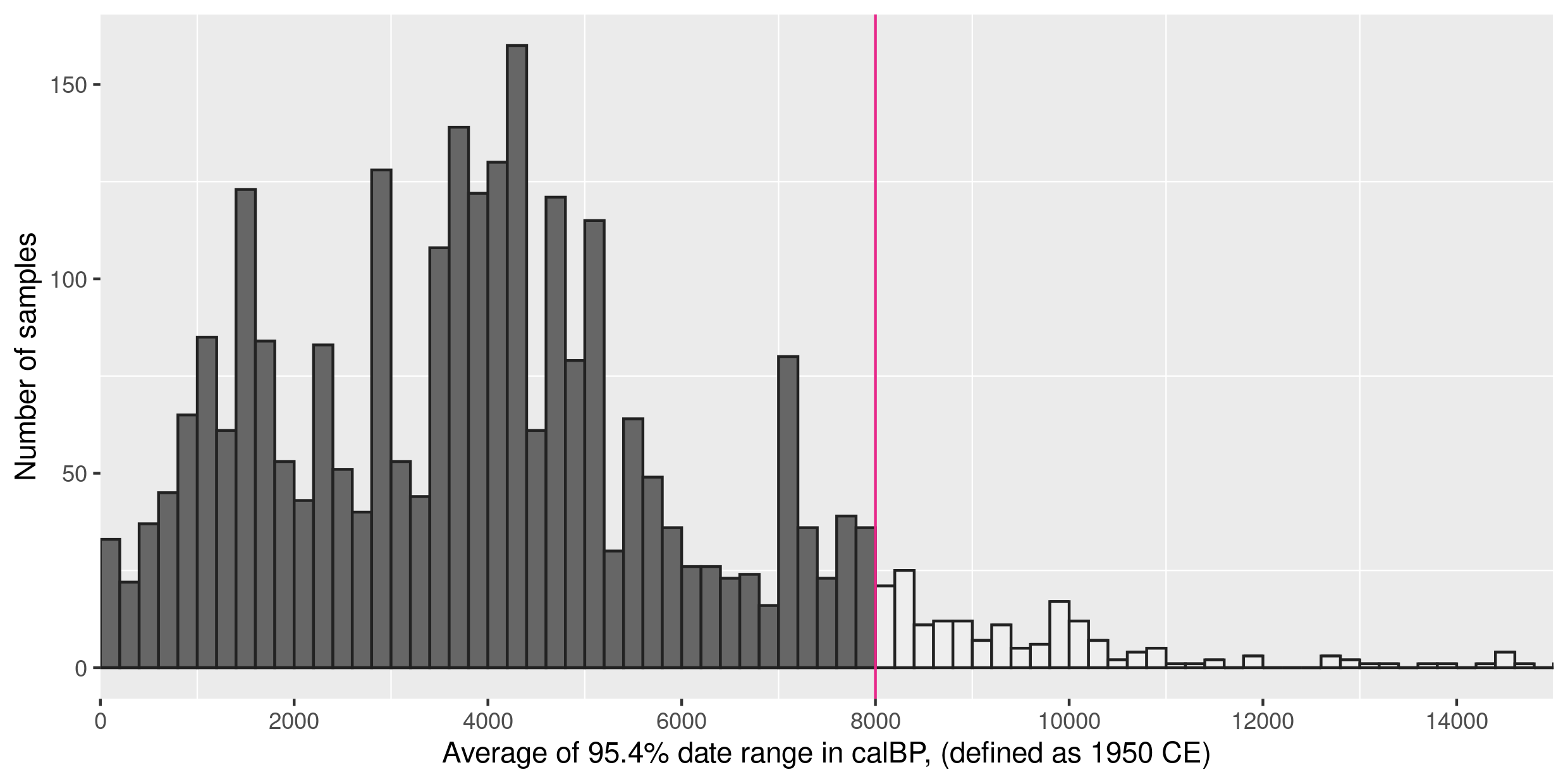


**Figure S4.** The date distribution of 2809 ancient individuals from the V42.2 AADR 1240K data set (handful of extremely old, >15000 years) samples were excluded from the plot for better visualization. Since kinship analysis requires proper reference population we excluded 216 individuals older than 8000 calBC from further analysis.


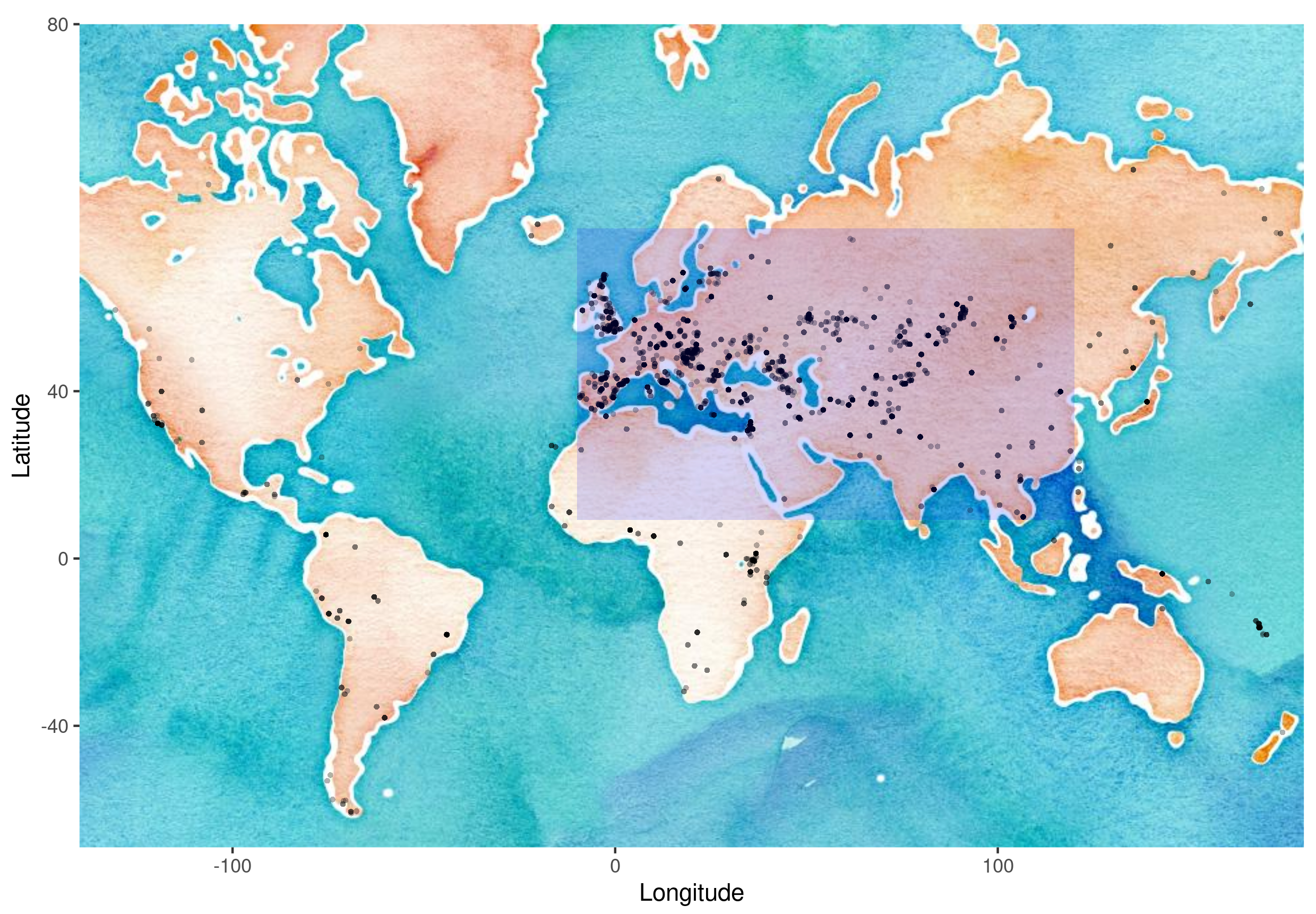


**Figure S5.** The geographical distribution of the samples of 2594 ancient individuals from the AADR 1240K dataset. As kinship analysis depends on proper reference populations we excluded 458 individuals from poorly represented geo locations resulting 2136 ancient individuals included in the final data set.
