# Supplementary material for: correctKin: an optimized method to infer relatedness up to the 4th degree from low-coverage ancient human genomes
Source: Genome Biol. 2023 Feb 28;24:38. doi: 10.1186/s13059-023-02882-4 (PMC9972692; doi:10.1186/s13059-023-02882-4)
Supplement: Supplementary file 8 — Additional file 8: Note S1. Installation requirements and a step-by-step method description including detailed usage examples to perform correctKin analysis from various data sources. [file 13059_2023_2882_MOESM8_ESM.docx]

Supplementary Note

Table of content

[*Software requirements* 1](#_Toc100031924)

[*Random allele calling of ancient samples with ANGSD from BAM files* 1](#_Toc100031925)

[*Importing data from VCF files of modern samples.* 3](#_Toc100031926)

[*Using diploid PLINK data with the 1240K marker set* 3](#_Toc100031927)

[*Using haploid PLINK data with the 1240K marker set* 3](#_Toc100031928)

[*Performing PCangsd on a PLINK data set* 3](#_Toc100031929)

[*Calculating the marker overlap fraction between samples* 4](#_Toc100031930)

[*Filtering the kinship coefficient matrix for relatives* 4](#_Toc100031931)

[*Practical workflow for analyzing ancient/low coverage WGS data* 5](#_Toc100031932)

# *Software requirements*

Linux OS with standard shell tools (sed, awk, etc) and developer tools (gcc, make, etc).

Basic genome data manipulation (plink2): <https://www.cog-genomics.org/plink2>

Random allele calling from BAM files (angsd): <http://popgen.dk/angsd/index.php/ANGSD>

Kinship coefficient estimation (PCangsd v.0.99): <https://github.com/Rosemeis/pcangsd/releases/tag/v.0.99>

Correction of kinship coefficient for low coverage data: <https://github.com/zmaroti/lowcovkin>

# *Random allele calling of ancient samples with ANGSD from BAM files*

**Note:** this methodology is suitable for low coverage shallow WGS forensic data as well when diploid calling is not available due to the amount of biological sample, or when at least one of the samples are low coverage. **Do not mix diploid and pseudo haploid data** as it will be a much bigger factor in PCA than real allele frequencies.

**1) preparing a sites file for restricting variant calling to 1240K autosomal SNPs**

# getting the AADR 1240k marker set

wget <https://reichdata.hms.harvard.edu/pub/datasets/amh_repo/curated_releases/V42/V42.4/SHARE/public.dir/v42.4.1240K.snp>

This is the same as the actual V50.0 data set's 1240K ‘.snp’ file. For kinship analysis we need to restrict the analysis to the autosomal positions only.

# the EIGENSTRAT ‘.snp’ file format:

# rs3094315 1 0.020130 752566 G A

# get column 2,4 and print autosomal sites only

sed -r 's/^[ ]+//;s/[ ]+/\t/g' v42.4.1240K.snp | cut -f 2,4 | awk '{if($1>=1 && $1 <=22){print}}' >v42.4.1240K_autosomal.sites

# indexing sites file before using with ANGSD

angsd sites index v42.4.1240K_autosomal.sites

Index preparation has to be done only once, and the resulting sites file can be used in all analyses where the same marker set is used for the analysis.

**2) random allele calling with angsd**

# creating list of BAM files

ls *.bam >BAM_LIST.txt

# parallel haploid calling with ANGSD

parallel -j 22 angsd -doHaploCall 1 -sites 42.4.1240K_autosomal.sites -r {} -doCounts 1 -bam BAM_LIST.txt -nThreads 1 -out GT_1240_{} :::: <(seq 1 22)

The result is 22 files with name of *GT_1240_(1-22).haplo.gz* containing the pseudo haploid random allele call genotypes of the samples provided in the *BAM_LIST.txt* file. ANGSD automatically assigns “ind0”, “ind1”, … names for samples 1-N found in the bam list file. As a matter of fact, one can run ANGSD with less thread (-j N flag with the parallel software) in case less CPU cores are available or skip the use of parallel altogether:

# random haploid calling without parallel scatter gather

angsd -doHaploCall 1 -sites 42.4.1240K_autosomal.sites -doCounts 1 -bam BAM_LIST.txt -nThreads 1 -out GT_1240

In this case we will get all our genotypes in one file: GT_1240.haplo.gz

*Importing ANGSD pseudo haploid data*

# importing data from multiple ANGSD “.haplo.gz” files (parallel call)

importHaploCall 42.4.1240k.snp GT_1240_haploid.bed GT_1240_*.haplo.gz

# importing data fom a single ANGSD “.haplo.gz” file

importHaploCall 42.4.1240k.snp GT_1240_haploid.bed GT_1240.haplo.gz

In both cases the output file is a binary PLINK data file and the corresponding marker file (GT_1240_haploid.bed, GT_1240_haploid.bim) that have the genotypes in SNP major format based in the provided EIGENSTRAT.snp file’s major/minor SNPs. All conflicting SNPs that do not correspond to the expected major/minor are logged in the appropriate indX.log file and these markers are set as “unknown” genotype in the resulting PLINK data set. Since ANGSD “.haplo.gz” files have a general “ind0”, “ind1”, … naming convention for the samples provided in the BAM_LIST.txt the family information (FAM) file has to be prepared manually with the proper sample IDs, sex (inferred from other analysis) and population IDs. The provided EIGENSTRAT.snp is used to create the PLINK “.bim” file with the appropriate marker data.

**Note**: in case the output file ends with a “.geno” suffix, then a plain text EIGENSTRAT data set is created (with the corresponding .snp file). In this case naturally, the corresponding “.ind” file has to be created manually.

# *Importing data from VCF files of modern samples.*

**Note:** This is the most “risky” data format to use for this analysis when using data from various sources. The best result is achieved when starting from a JOINT genotype call of several samples (i.e. joint VCF from the GATK pipeline). Individual VCF files may have HOM REF at positions where no genotype is called or they can have NO COVERAGE meaning missing data. If data is generated from proper high coverage WGS it can be assumed that all positions are covered and no GT called in and individual VCF file at a particular position means HOM REF allele. If we have high quality data, and the variants are QC filtered, then all variants with higher >0.05 MAF can be used for the analysis when proper reference populations are available (for example 1KG Phase 3 data) and the 1240K marker set is not necessary. However, this will restrict the analysis for the given sample set and likely only the curated 1KG Phase III data as source of reference population. When using individual VCF files, or data from different laboratories for better comparability it is recommended to restrict the analysis to the high frequency, biallelic marker set (for example the AADR 1240K marker set).

Keep in mind, plink does not use the reference sequence and the genotype of alleles stands for minor and major alleles, that can be different between data sets for any markers (depending on the included individuals and their particular genotypes at each marker position), thus even when genotypes are restricted to the 1240K sites it is not guaranteed that major and minor alleles in the given data set will equal the REF/ALT alleles provided in the AADR data set “.snp” file. Consequently, these samples can be only analyzed against the samples imported together:

plink --vcf JOINT.vcf --make-bed --chr 1-22 --double-id --a2-allele JOINT.vcf 4 3 '#' --out GT_1240_diploid

# *Using diploid PLINK data with the 1240K marker set*

# for kinship analysis data has to be restricted to the autosomal chromosomes:

plink –bfile GT_1240_diploid_all –make-bed –chr 1-22 -keep-allele-order –out GT_1240_diploid

**Note:** to avoid PLINK “automagically” flip/change minor/major always use the “-keep-allele-order” option when transforming/merging data with PLINK.

# random pseudo haploidizing PLINk data set

pseudoHaploidize -out GT_1240_haploid GT_1240_diploid.bed

**Note:** If all data are high quality diploid there is no need to apply this step as PCangsd can work with both diploid and pseudo-haploid data while diploid data results in less technical error. However, if any of the samples are low coverage then comparison can be only done if the whole data set is pseudo haploidised. **Do not mix diploid and pseudo-haploid data** in kinship analysis as it will be the major factor differentiating samples in PCA instead of the allele frequencies.

# *Using haploid PLINK data with the 1240K marker set*

# for kinship analysis data has to be restricted to the autosomal chromosomes

plink –bfile GT_1240_haploid_all –make-bed –chr 1-22 -keep-allele-order –out GT_1240_haploid

**Note:** to avoid PLINK “automagically” flip/change minor/major always use the “-keep-allele-order” option when transforming/merging data with PLINK.

# *Performing PCangsd on a PLINK data set*

**Note:** if all data is high quality diploid, it is preferable to perform the analysis with diploid data. However, when there is at least one sample with low coverage, partially genotyped and/or genotype accuracy is questionable it is preferable to transform the diploid data set to haploid by the “pseudoHaploidize” tool. **Do not mix diploid and pseudo-haploid data** as it will be a much bigger factor in PCA than real allele frequencies.

The current release of PCangsd does not include the option to perform kinship analysis, thus we need to download and install the v0.99 version of the software:

wget <https://github.com/Rosemeis/pcangsd/archive/refs/tags/v.0.99.tar.gz>

When installed to the given PATH the analysis can be performed with the following command:

python PATH/pcangsd-v.0.99/pcangsd.py -plink GT_1240_haploid -o GT_1240_haploid -inbreed 1 -kinship -threads N

Replace “--threads N” with the apprpriate amount of threads to speed up the calculation.

The analysis will result in a plain text file (*GT_1240_haploid.cov*) containing the covariance matrix and two binary files (*GT_1240_haploid.inbreed.npy,* *GT_1240_haploid.kinship.npy*) containing the inbreeding coefficients of the analyzed samples and the estimated kinship coefficient matrix in *numpy* format.

# *Calculating the marker overlap fraction between samples*

We defined marker overlap fraction between two samples as the number of genotyped markers that are genotyped in both samples divided by the number of markers in the data set. Thus it is a number in the range of 0 and 1.

# to calculate the pairwise marker overlap fraction, use the following command:

markerOverlap GT_1240_haploid.bed

The output is a file *GT_1240_haploid.overlap* that contains the pairwise marker overlap fraction matrix between all sample combinations.

**Note:** PCangsd by default filters the SNPs by MAF>=0.05 to remove very rare alleles and markerOverlap will also consider only the markers that are MAF>= 0.05. In case you use a different MAF threshold for PCangsd, also apply the “-maf MAFTRESHOLD” when running markerOverlap.

# *Filtering the kinship coefficient matrix for relatives*

# flat output of relatives

filterRelates PREFIX.kinship.npy PREFIX.overlap >PREFIX.rels.tsv

The *filterRelates* tool will read the output of the PCangsd estimated kinship coefficient matrix (PREFIX.kinship.npy) and the overlap fraction matrix (PREFIX.overlap) calculated by *markerOverlap* tool. Based on these two matrices it calculates the pairwise corrected kinship coefficient between all individuals. The hypothesis is that between the relatives most of the kin relations are “unrelated”, thus the standard deviation of the corrected kinship coefficient is the technical error of the whole workflow (this is true in most cases especially if we have a reference population with sufficient number of individuals). By default, for bins of overlap fraction ranges the tool calculates the 6 sigma threshold deviation from the mean of corrected kinship coefficient (see Figure 5 in the main manuscript) and uses this threshold to filter out unrelated kin relations. The tool outputs two files: PREFIX.corr.tsv a plain TAB separated text file containing the corrected kinship coefficients in the upper triangle and the original estimated kinship coefficients in the lower triangle of the matrix and PREFIX.stats.tsv a plain TAB separated text file containing statistics on the bins of marker overlap range (min, max, median), the N sigma threshold, confidence interval of corrected kinship coefficient of the bin. Based on the threshold the tool outputs all kin groups (groups of related individuals) in a flat TAB separated format to the STDOUT. The columns of the output contains the following information:

GROUPID number of the kin group

ID1 sample ID 1

ID2 sample ID 2

uncorrKinCoeff the estimated kinship coefficient between the two samples

overlapFrac overlap marker fraction between the two samples

corrKinCoeff the corrected kinship coefficient between the two samples

N sigma Thresh the N sigma threshold of the overlap bin (containing ovelapFrac)

95% conf (lower) 95% confidence interval lower limit of the corrected kinship coeff

95%conf(upper) 95% confidence interval upper limit of the corrected kinship coeff

estimated Relatedness estimated relatedness (with text)

In case the corrected kinship coefficient is above the selected (N sigma) threshold the mapping of the corrected kinship coefficient to the most likely degree of relation is done using the following kinship coefficient thresholds. The lower bound is the mean of the kinship coefficients corresponding to the given degree and one-degree higher relation, while the upper bound is the mean of expected kinship coefficients corresponding to the given degree and one-degree lower relation. Accordingly, we used the [0.1875, 0.375], [0.09375, 0.1875], [0.046875, 0.09375] and [0.0234375, 0.046875] bounds as thresholds for to 1^st^, 2^nd^, 3^rd^ and 4^th^ relations.

Alternatively, the tool can output the sub matrices of kinship coefficients of the identified kin groups as a TAB separated flat text file.

# matrix output of relative’s kinship coefficients

filterRelates –matrix PREFIX.kinship.npy PREFIX.overlap >PREFIX.rels.coeffmat.tsv

**Note**: *filterRelate* has options for hard filtering based on corrected kinship coefficients, or using hard/sigma threshold (whichever is higher), using different sigma threshold or filtering for preset IDs with or without kinship coefficient thresholds. For the full documentation of the options read the README of *filterRelate*.

# *Practical workflow for analyzing ancient/low coverage WGS data*

# creating list of BAM files

ls *.bam >BAM_LIST.txt

# random allele calling with ANGSD using the prepared sites file

angsd -doHaploCall 1 -sites 42.4.1240K_autosomal.sites -doCounts 1 -bam BAM_LIST.txt -nThreads 1 -out GT_1240

# importing ANGSD haploid call output into PLINK binary data set

importHaploCall 42.4.1240k.snp GT_1240_haploid.bed GT_1240.haplo.gz

**Note**: the corresponding “.fam” file of the PLINK data set has to be prepared manually (including sex determination from other analyses and the proper sample IDs that are not contained in the ANGD output files).

# performing kinship analysis with PCangsd v0.99

python PATH/pcangsd-v.0.99/pcangsd.py -plink GT_1240_haploid -o GT_1240_haploid -inbreed 1 -kinship

# calculating the pairwise marker overlap fraction matrix of samples

markerOverlap GT_1240_haploid.bed

# using the estimated kinship coefficient matrix and marker overlap fraction matrix to calculate the corrected kinship coefficient matrix, statistical analysis of corrected kinship estimates, and filter relatives

filterRelates GT_1240_haploid.kinship.npy GT_1240_haploid.overlap >GT_1240_haploid.relatives.tsv

Note: the last command will also output GT_1240_haploid.corr.tsv and GT_1240_haploid.stats.tsv (see documentation on *filterRelates*).
