# Supplementary material for: correctKin: an optimized method to infer relatedness up to the 4th degree from low-coverage ancient human genomes
Source: Genome Biol. 2023 Feb 28;24:38. doi: 10.1186/s13059-023-02882-4 (PMC9972692; doi:10.1186/s13059-023-02882-4)
Supplement: Supplementary file 9 — Additional file 9. Review history. [file 13059_2023_2882_MOESM9_ESM.docx]

Review History

**First round of review**

**Reviewer 1**

**Were you able to assess all statistics in the manuscript, including the appropriateness of statistical tests used?** No

**Were you able to directly test the methods?** No

**Comments to author:**

Nyerki et al. propose an approach based on the PC-Relate method for inferring degrees of relatedness of up to 4th degree in low coverage ancient human genomes. This is an important problem that merits more methods development and so it is nice to see this work. A challenge with the current proposed method is in its evaluation. To simulate ancient DNA (aDNA) most realistically, one should ensure that: the number of typed markers is similar to that achievable in ancient samples; the effects of degradation and possibly contamination are mimicked (and therefore some erroneous genotype calls are included); and the effects of inbreeding and/or increased drift that can be present in ancient populations are characterized. The current study does not incorporate these considerations as fully as would be ideal - it uses modern data and down samples to lower coverage while retaining only one allele (i.e., pseudo-haploidizing the data). The down sampling to 5% overlapping markers would correspond to a pair of ancient samples with coverage of roughly c1 * c2 = 0.05. It would be good to have a discussion of this in the text and to know what proportion of aDNA pairs have this level of overlap. What happens to the kinship estimates if the authors include genotyping errors? Is 5% overlap the minimum recommended for using this new approach?

How many samples from the same (or very similar) population are needed to run the PCA? This would be good to understanding as many aDNA populations have few samples available. If the idea is to use modern samples as references (please say if this is the case), a much more robust set of analyses to ensure this will work or a description of the settings in which it would work are needed. I was confused by the 1000 Genomes super-population analysis and how this fits in with the aDNA applications highlighted in this work. Notably, the Discussion says that using the super-population resulted in “comparable although slightly higher kinship coefficients”. This bias, when present at low coverage, could be amplified. As such, might samples have their degree of relatedness overestimated? As the paper talks about having a “proper reference population”, a more explicit description of how to obtain this for aDNA samples is needed.

Along these lines, one thing that would be good to include is a more explicit description of how the method works, particularly the PCA analysis. Even though it’s based on work in a published paper (PC-Relate), describing the pipeline and an intuition of how the PCA impacts the results - together with the potential biases this could introduce if it were not run on a large enough sample, for example - would be very useful. Figure 6 gives a workflow, but perhaps I missed the where the PCA analysis fits in.

It’s OK to keep thing as they are, but the authors may wish to reduce the analyses that have limited down sampling and/or no errors. The most relevant figures are 4 and 5, while Table 1 is not so realistic for aDNA and could be moved to the supplement. Additionally, a discussion of the (very sensible) correction factor for marker overlap fraction could come sooner such that Figures 1 through 3 could depict the corrected kinship coefficients.

Use of more robust statistical evaluations is something that would also be good to see. There are small numbers of simulations instead of dozens. With a larger simulation set, the authors could calculate a mean and standard deviation of the kinship coefficients for different coverage percentages, which would help solidify the efficacy of this approach.

Finally, for the real data analysis in the REICH dataset, it would be good to include the READ and 1cMLkin results. This may reveal some of the new approach’s limitations and strengths.

Minor:

In Figure 2, do the authors know why there is a discontinuity at marker overlap fraction of 60%?

In Figure 5, the text indicates this ranges down to about 10% marker overlap, but the plot seems to include values below 10%. Along the lines of the comments above, what percentage of marker overlap is feasible for this method to operate on? Should the plot go down to lower values?

Detailed comments:

- Line 16 and 44: “deeply sequenced modern DNA” and “deeply typed WGS data” - note that the samples can also be SNP array genotyped.

- Line 18-9 and 336-7: “has been possible up to the second degree with large uncertainties” and “only first and second degree relations can be detected” - but note that 1cMLkin identifies relatives of 3rd to 5th degree, so rewording this seems necessary.

- Line 38: “including GERMLINE, fastIBD, GRAB, ANGSD” - this isn’t the most recent list of methods and while that’s OK, the authors could cite Ramstetter et al. (Genetics, 2017) (which also may not be ideal since there are several even more recent methods this doesn’t consider).

- Lines 45-46: “an appropriate set of reference data” - note that not all algorithms explicitly (or even implicitly) use a reference set. KING and IBIS, for example, only analyze the genotypes of each sample pair to quantify their relatedness.

- Although the analyses as given may give an adequate picture, it would be better to do more than three simulations (Table 1). Doing several dozen would allow the authors give a mean and standard deviation (for example).

- To help readers, it would be helpful if the number of SNPs included in each analysis was given in the caption. That is, for analyses where no down sampling (or down sampling to 1240k), please state this in the captions (e.g., Table 1).

- Line 244-5: “represents 6 sigma threshold” - is this for unrelated pairs?

- Line 256-7: “the analysis shows that the correction itself does not significantly multiply the error rate” -- this may be a bit too strong. Perhaps including more pairs and giving standard errors for the kinship coefficients of pairs with known relatedness would make this statement easier to back up with data.

- Line 304-5: “we filtered potential relatives above 0.046875 corrected kinship coefficient” - what is the reason for this filter?

- Line 353: “lead to major bias” - this could be right. Are there any papers that show this? If not, perhaps being a little less certain in the wording (and explaining the reasoning) would be good.

- Line 372: “as used in prior protocols” - perhaps citing here would help?

- Line 412: “our approach could properly identify all relations up to 4th degree” - but how do we know what the true relationship is here?

- Figure 6: I wasn’t totally sure I knew what happens at each stage of this workflow. For example, is PCA calculated by PCAngsd? Adding to the caption and to the Methods (or other) sections would help specify the way the approach works. On this figure, what is the difference between the curated PLINK dataset and fully typed/modern samples?

**Reviewer 2**

**Were you able to assess all statistics in the manuscript, including the appropriateness of statistical tests used?** Yes

**Were you able to directly test the methods?** No

**Comments to author:**

Nyerki et al describe some observations about the performance of PC-relate with regard to low-coverage data. They use the knowledge from these observations to implement a normalization scheme that greatly increases the sensitivity of this program for detecting more distant relations. The design and description of their work is adequate. Helpfully, they analyze the large REICH dataset, detecting many instances of apparent sample mislabeling.

This is a nicely conceived and executed piece of work. I have several suggestions that the authors may mind helpful, listed below.

1. The central observation is that kinship coefficient calculated by pcrelate is strongly, linearly correlated with the number of markers available in the comparison. This observation is demonstrated very clearly. This observation, however, may simply be described as a bug in pcrelate. It seems as though the authors of pcrelate may not have contemplated the case of an incomplete dataset and simply do not divide some number by the appropriate denominator. In any case, once this result is shown (Figure 1), it does not add much to the paper to include the "marker overlap fraction" to all subsequent figures. The reader will get this simple point from the authors' clear explanation and the results of figure 1. Consider simplifying the subsequent figures in this way.

2. The figures, in general, are not well labeled. The authors rely on putting information in the figure captions. A well-designed figure does not require much of a caption as all the labeling is in the figure itself. In particular, Figure 2 should have the 1000Genomes population labels included in the legend of the figures. For example: GBR (HG00124-HG00119; 2nd degree)). Also, in Figure 3, the axis labels in Panel a and b are identical. One must read the caption to learn how they differ. Why not put that information in the figure itself?

3. The analysis of the mixed ethnicity Hungarian cohort showed that using allele frequencies from multiple panels "fixed" the problem of using the wrong population. This suggests that a better approach for some (maybe all?) samples would be to use a more general population model. The authors should test their method using all of 1000Genomes as the "population". They may find that the wonky behavior of using the "wrong" population is fixed by simply using a general, comprehensive model of "human" allele frequencies as the background PCA model.

4. Line 17, "population" should be "populations"

5. The second paragraph of the background alternates between a general description of the problem to specific minutiae of their implementation (using only bi-allelic, high minor-allele frequency SNPs). This section could be better written with shorter, more coherent paragraphs.

6. Line 116 "where 50-50%" is unclear

7. The sentence in Lines 133-135 seems to be the central "normalization" done to fix what might be described as a bug in pcrelate. As written, this seems to be a minor tweek. In fact, it's the main insight! As written, the reader may not appreciate how simple the solution is. Consider contextualizing this statement appropriately.

8. Figure 2, panel b has an interesting anomaly around marker overlap fraction 60. The authors may want to speculate why the trend of kinship coefficient is discontinuous through this region of marker overlap fraction.

9. In figure 5, there are many sample comparisons that appear to be 1st, 2nd and 3rd degree relatives. Are these known relationships? That is, should these be considered positive controls or errors? In either case, they should be labeled and discussed.

10. Line 335, the phrase "Fst is very close…" should be changed to "Fst is very low"

11. The authors may care to comment on the following suggestion: Perhaps their method would be more robust to population knowledge if the first pre-selected only markers that have low Fst between populations. In this way, it may not be necessary to know, a priori, what the appropriate background model should be.

12. Line 434 "…already fixated at this age". I assume the authors mean "…already fixed at this age", i.e., were at 100% population frequency and not segregating variation. In any case, that is unlikely to be true since fixed sites should be less informative in this analysis.

13. In general, the discussion is a bit tedious. It is mostly a rote enumeration of the results. It should be shortened, focusing on contextualizing the results.

14. The authors are to be commended for their efforts to detect and describe sample mix-ups in the Reich dataset.

**Authors Response**

**Point-by-point responses to the reviewers’ comments:**

Reviewer #1: Nyerki et al. propose an approach based on the PC-Relate method for inferring degrees of relatedness of up to 4th degree in low coverage ancient human genomes. This is an important problem that merits more methods development and so it is nice to see this work.

Thank you very much for the constructive criticism of our work and the valid points raised. Based on your feedback and the additional simulations performed, we could better present the strengths and potential limitations of our methodology.

A challenge with the current proposed method is in its evaluation. To simulate ancient DNA (aDNA) most realistically, one should ensure that: the number of typed markers is similar to that achievable in ancient samples; the effects of degradation and possibly contamination are mimicked (and therefore some erroneous genotype calls are included); and the effects of inbreeding and/or increased drift that can be present in ancient populations are characterized. The current study does not incorporate these considerations as fully as would be ideal - it uses modern data and down samples to lower coverage while retaining only one allele (i.e., pseudo-haploidizing the data). The down sampling to 5% overlapping markers would correspond to a pair of ancient samples with coverage of roughly c1 * c2 = 0.05. It would be good to have a discussion of this in the text and to know what proportion of aDNA pairs have this level of overlap.

In our simulation of down sampled modern samples we used similar marker counts that were present in the 2136 selected individuals of the experimental REICH aDNA data set. It is typical that very low genome coverage or questionable aDNA samples are excluded from admixture, PCA, qpAdm and other population genetic analyzes. The very low endogenous DNA content (<0.1x genome coverage) „over sequenced” samples may contain significant exogenous DNA contamination while the low marker count samples could decrease the resolution of many population genetic analyzes. Accordingly we used a marker count that is roughly equivalent with this 0.1x genome coverage limit. Statistically 0.1x genome coverage means that approximately 10% of the genome is covered (in case we take that at this low coverage majority of genome positions are covered a single read). In this case it means that roughly 10% of the whole marker set is genotyped. In the used 1240K data set it would be equal to roughly ~115K autosomal markers. We used 100000 markers as the lower limit, thus in this simulation c1 * c2 could be as low as ~0.00755 meaning less than 1% marker overlap between such low coverage sample pairs.

In the experimental aDNA analysis (Kinship analysis of ancient samples from the REICH 1240K data set) there are 169467 sample pairs (3.72%) where the pairwise marker overlap fraction is less than 0.05 percent and 1936 cases (0.04%) where it is less than 0.01 percent.

In our statistical analysis of modern samples (Statistical validation, assessment of technical errors) we had similar figures as of the experimental aDNA data set. We down sampled the modern samples between marker count 100.000 to 1.2M corresponding to the aDNA samples marker counts. Accordingly, in the actual marker overlap data there are ~36000 sample pairs (3.46%) where overlap is less than 0.05 percent (<57000 markers overlap between two samples) and 326 cases (0.03%) where overlap is less than 0.01 percent (<11500 markers overlap between two samples.

As suggested we incorporated this information into the Discussion section:

“In the statistical evaluation using the down sampled modern diploid/pseudo haploid data we simulated marker counts similar to aDNA data. We applied the same minimum 100000 genotyped markers per individual threshold that was used in the analysis of 2136 selected ancient individuals from the AADR dataset. This equals roughly 0.08x genome coverage considering the ~1.15M autosomal markers of the 1240K marker set. Thus the simulated data had similar marker counts and distribution as the analyzed AADR dataset. Accordingly, pairwise marker overlap was <5% (<57000 markers) between 3.72% and 3.46% of the analyzed sample pairs (ancient and modern respectively).”

In the original PC-Relate manuscript they state that “*The PC-Relate estimator ϕˆij presented in Equation* [*4*](https://www.ncbi.nlm.nih.gov/pmc/articles/PMC4716688/#fd4) *is still an appropriate estimator of the kinship coefficient due to recent pedigree structure in inbred populations.” and their simulations (Performance in inbreed populations) seemed to support this: „PC-Relate provided consistent kinship coefficient estimates with low variability, even in the presence of inbreeding. In contrast, KING-robust provided consistent estimates only for pairs of individuals who were both outbred and from the same subpopulation (*[*Figure S14*](https://www.ncbi.nlm.nih.gov/pmc/articles/PMC4716688/#mmc1)*).*”. However they also note that some of their allele sharing probability estimators could be worked on and caution should be taken for analyzing inbreed populations.

In case of small drifting populations, we think that the analysis could suffer only when lacking the appropriate number of proper reference individuals, however this limitation would likely affect other kinship estimators as well that are based on a presence of suitable reference population and allele frequencies.

According to the raised concerns we added the sentences: „ Furthermore, while PC-Relate kinship coefficient estimator is known to be appropriate even in inbreed populations [24], we have to caution that in case of inbreed or small drifting populations extra care has to be taken to confirm that the test individuals are analyzed with their own reference population.” to the Discussion section.

What happens to the kinship estimates if the authors include genotyping errors?

We performed simulations (Supplementary Table 2) to test the effect of typical aDNA related genotyping errors (post mortem damage, exogenous DNA contamination, endogenous DNA contamination and mixed contamination). We included the description of the applied methods, results and the conclusion in the manuscript showing that our method can be safely used when genotyping errors are not considerably higher than in typical aDNA data:

Accordingly, we added two new sections in the manuscript to describe our simulations and results (RESULTS/*The effect of genotyping errors on the corrected kinship coefficient;* METHODS/*Simulation of aDNA related genotyping errors).*

We also added a new paragraph in the Discussion section: “Genotyping error simulations show that approximately double error rate compared to typical experimental aDNA data leads to 5.4-10.4% proportionally lower corrected kinship coefficient than the expected kinship coefficient in our workflow (Figure 2, Supplementary Table 2). The mean corrected kinship coefficient of the validated sample dups and 1st relatives of the experimental 2136 AADR individual was 0.48 and 0.24 respectively (approximately ~4% lower from the expected). This is in accordance with the mean X-contamination rate (1.28%) of ancient individuals of the AADR V42.2 dataset suggesting that our kinship-estimation method can be safely used on typical aDNA data. Nevertheless, analysis of highly contaminated (CRITICAL/FAIL) samples containing higher rate of genotyping errors (>5%) could lead to underestimation of corrected kinship coefficient and as a result to underestimation of the degree of relation especially in case when the relatives share less than the expected IBD fragments due to true biological variation.”

Is 5% overlap the minimum recommended for using this new approach?

The 5% overlap is not a minimum recommendation, in fact we identified 80 relatives bellow this overlap fraction from the experimental REICH aDNA data.

The 5% overlap fraction was solely used as a sensible step to do time consuming simulations and analyze the effect of marker overlap on the estimated kinship coefficient in the whole workflow.

The overlap fraction is a metric to correct the estimated kinship coefficient in sparse partially genotyped data set. However, the statistical power of the analysis not only depends on the actual absolute pairwise marker counts between samples, but also on the level of contamination or the availability of proper reference population as revealed by our results. Thus we cannot offer a single number as a recommendation as the quality of data or the number of markers could be largely different in the analyzed data sets. However the “filterRelates” tool (included in our toolset) uses the experimental error model of the analyzed data set to distinguish between unrelated and potentially related people. Thus in case at the given overlap fraction the estimated kinship coefficient is above the 6 sigma threshold it will be identified as a potential kin, regardless if the overlap fraction is below or above five percent.

How many samples from the same (or very similar) population are needed to run the PCA? This would be good to understanding as many aDNA populations have few samples available. If the idea is to use modern samples as references (please say if this is the case), a much more robust set of analyzes to ensure this will work or a description of the settings in which it would work are needed.

In general always the best reference population is the same reference population as the test individuals. In case aDNA samples exist they should be used, if not but a modern population have very similar genome composition to the analyzed ancient samples the modern data can be used as a reference. This is exactly the case at the analyzed CWC samples. According to our results (Table X, Supplementary Table X) the low coverage (0.12x-4.18x) ancient CWC individuals (2500 BCE) gave proper, almost the same corrected kinship coefficients (up to 4th degree) using the 1KG EUR reference pop or the mixed set of 2136 REICH ancient individuals (including 25 CWC samples) as reference.

Unfortunately there is no simple answer to the “How many samples are needed…?” question. As ultimately it means how many individual contains a sufficiently large subset of the variations representing the whole population (to allow distinguishing between IBS and IBD). This number would be surely different for homogenous stable, small drifting, or heterogenous multiple admixed large populations.

In our performed analyses from modern data 50-100 individuals were sufficient. We also have to note that the genome structure of modern populations are much more complex compared to ancient (especially the older pre iron age) samples. They contain more admixture components and accordingly their heterogeneity is described by higher number of relevant eigen vectors in PCA. Thus we would expect that in case of modern samples more individuals are needed to represent all admixture components (even the minor ones) and the proper allele frequencies of component specific markers of the underlying population structure.

As a side note, we could also ask how to decrease the uncertainty that we miss important population components from the reference that is present in our test individual? Since our method scales very well on larger data sets we think that “the more is better” approach could be applied when there are suitable number of individuals since we could likely represent all population specific markers better.

To call the attention of the readers to these concerns we added the sentences „ According to our analysis ~50-100 unrelated individuals are sufficient as a reference in case of modern samples. We speculate that in case of populations with less complex genome structures (like pre iron age populations) a smaller number of unrelated individuals could likely represent the population structure properly. This is also demonstrated in case of the validated relations of the analyzed CWC individuals where the analysis resulted comparable kinship coefficient estimates using modern EUR individuals as a reference or the 25 Czech, Latvian, Estonian and German CWC individuals of the AADR dataset (Table 2, Supplementary Table 5).” in the discussion.

I was confused by the 1000 Genomes super-population analysis and how this fits in with the aDNA applications highlighted in this work. Notably, the Discussion says that using the super-population resulted in “comparable although slightly higher kinship coefficients”. This bias, when present at low coverage, could be amplified. As such, might samples have their degree of relatedness overestimated? As the paper talks about having a “proper reference population”, a more explicit description of how to obtain this for aDNA samples is needed.

In the first part of the manuscript we wanted to dissect the various factors (random pseudo haploidisation, marker overlap, reference population, population structure /simple or admixed/) separately that could influence or bias the kinship coefficient estimation in our whole workflow. For this we used fully typed modern samples with known relations and known genome structures. We did not use aDNA for this work as these factors could not be controlled well. For example not many aDNA is available where we have full diploid calls so we can compare diploid, haploid data or perform the marker overlap simulations on the whole range of overlap fractions. Furthermore in case of public data we cannot control the genotyping quality and all the additional factors (contamination, PMD) present in aDNA that also alters the outcome of the analysis. Since there are no proven distant relatives in public aDNA samples we could not test how the estimated kinship coefficient depends on the analyzed factors in case of known distant 4-5th relatives.

We had shown (Figure 4, Supplementary Figure 2, Supplementary Table 3) that the correction does not amplify the reference bias, in this particular case regardless of the correction statistically we would get the same corrected estimated kinship coefficient as we get from the fully typed diploid data set. Thus it is clearly a reference problem, nevertheless it is a limitation that we have to be aware of. Accordingly we elaborated on this in the discussion:

“On the other hand, using the samples’ super-population as reference resulted in comparable although slightly higher kinship coefficients compared to the proper reference population (Supplementary Figure 2) proving the robustness of the PC-Relate algorithm. This reference bias is not amplified by the applied correction for marker overlap (Figure 4), however it could lead to the false identification of distant relatives.

We added a more explicit description how to obtain proper reference population: „When no prior knowledge exists on the reference population FST or FastNGSAdmix [32] analysis could be used as an objective method to select individuals best matching our test individual’s genome structure as a reference population.” to the Discussion to address this concern.

We also described the aim of our analysis more clearly by adding the sentence “In this analysis we wanted to investigate the scenario in which proper reference population is unknown or unavailable which is often the case for ancient samples.” to this paragraph in the result section.

Along these lines, one thing that would be good to include is a more explicit description of how the method works, particularly the PCA analysis. Even though it’s based on work in a published paper (PC-Relate), describing the pipeline and an intuition of how the PCA impacts the results - together with the potential biases this could introduce if it were not run on a large enough sample, for example - would be very useful. Figure 6 gives a workflow, but perhaps I missed the where the PCA analysis fits in.

In the original manuscript there are lots of deep mathemathics, however the principle of the analysis could be summarized with the sentences copied from the PC-Relate manuscript:

„*The PC-Relate kinship coefficient estimator is constructed using residuals from linear regression models that include PCs as predictors, and, therefore, the residuals are orthogonal to the PCs. As a result, ϕˆij measures genetic relatedness due to alleles shared IBD between i and j from recent common ancestors, because genetic similarities (or differences) due to more distant ancestry, as represented by the PCs in V, have been regressed out. Because the top D PCs completely capture the population structure in the sample, the expectation of* ***g****s conditional on* ***V*** *is equivalent to the expectation of* ***g****s conditional on the true ancestries of the sampled individuals. Therefore, the fitted values from this linear regression model can be used to predict individual-specific allele frequencies from the PCs.*”

Without the deep math we tried to summarize the essence of the PC-Relate methodology in the manuscript, and note the potential biases and included the sentences: „In the PC-Relate algorithm PCA is used to regress out the population specific IBS components. Using linear regression to fit individuals to the model all the remaining non regressed PC components are calculated as IBD. Thus insufficient amount of reference individuals, improper or missing population components in the reference or marker sets that are lacking informative markers of the tests lead to underestimation of IBS and inflated kinship coefficient estimation.” into the discussion.

The PCA analysis is the integral part of the PC-Relate kinship coefficient estimation. The PCAngsd implementation of the PC-Relate algorithm works with PLINK or EIGENSTRAT data format thus it can be easily used on the most comprehensive REICH aDNA data set. Since it is not a separate step that has to be performed we did not include an extra step in Figure 6. Since Figure 6. was confusing we remade it to differentiate the data import/shaping steps and also fixed the captions to display more clearly what’s going on at the different steps.

It’s OK to keep thing as they are, but the authors may wish to reduce the analyzes that have limited down sampling and/or no errors. The most relevant figures are 4 and 5, while Table 1 is not so realistic for aDNA and could be moved to the supplement. Additionally, a discussion of the (very sensible) correction factor for marker overlap fraction could come sooner such that Figures 1 through 3 could depict the corrected kinship coefficients.

Based on the feedback of all reviewers we restructured the manuscript and put the repetitive proofs that the applied data preparation techniques don’t distort the analysis into the Supplementary section (Supplementary Table 1-2, Supplementary Figures). Thus as suggested the equivalent of Table 1 was moved to the Supplements.

Accordingly, the most important marker overlap and the correction steps are presented first figures presented in the Results.

Use of more robust statistical evaluations is something that would also be good to see. There are small numbers of simulations instead of dozens. With a larger simulation set, the authors could calculate a mean and standard deviation of the kinship coefficients for different coverage percentages, which would help solidify the efficacy of this approach.

As suggested we performed additional simulations for all related individuals and sample dups presented in Supplementary Table 1 to assess the mean and SD of estimated kinship coefficients at different marker overlaps and random pseudo haploidization. We also visualized a subset of representative relations in Figure 1:

Figure 1. The mean and SD of the A) uncorrected and B) corrected kinship coefficient between selected 1KG individuals (sample dup, known 1st and 2nd degree) at different marker overlap fractions.

Our results demonstrate that the correction did not alter the mean of estimated kinship coefficient significantly. We modified the Results and Methods sections accordingly. We added a new Supplementary Table 1 containing mean/SD of simulations for all selected individuals.

We incorporated the sentences: „The results revealed that the kinship coefficient is a linear function of the marker overlap fraction. This allows a simple method for correcting the kinship coefficient value for low coverage genomes by dividing the estimated kinship coefficient with the marker overlap fraction between the two samples. According to our simulation the correction of estimated kinship coefficient for sparsely genotyped data resulted in reproducible kinship estimation regardless of the overlap fraction (Figure 1B). As expected low partially overlapping subset of markers would lead to less complete representation of the reference population and the test individuals thus regression of IBD/IBS based on the PC-Relate algorithm would have higher SD at low marker counts. Although the marker overlap fraction correction differs more than one magnitude between very low and high marker overlap fraction sample pairs, the analysis shows (Supplementary Table 1) that the correction itself does not significantly multiply the error rate (4x increase in SD at 20x correction factor) and the increased SD of the method are likely due to the higher uncertainty of PCA.” into the RESULTS/*The effect of overlapping marker fraction on the kinship coefficient calculation* section*.*

Finally, for the real data analysis in the REICH dataset, it would be good to include the READ and 1cMLkin results. This may reveal some of the new approach’s limitations and strengths.

Unfortunately, from our data set we cannot perform the lcMLkin analysis as it requires VCF files with proper genotype likelihood (GL) tags that is not available in the PLINK or EIGENSTRAT format. In a similar project we could not find any tool to convert REICH data to a proper VCF format that is accepted by the lcMLkin software and only the original BAM files and the python code supplied with lcMLkin was capable to generate the required VCFs. In case of these 2136 individuals we do not have the BAM files and collecting all the original data from various repositories would require enormous effort and time and large storage capacity.

Although due to data incompatibility we cannot assess the performance of lcMLkin on this data set, we have to note that in our settings the criteria was above 100000 out of ~1.2M genotyped markers that our lowest marker count equals less than 0.1x genome coverage. In the original (still preprint) lcMLkin manuscript the lowest genome coverage that was tested was 2x. In our analyzed aDNA dataset only 624 of the 2136 ancient samples have at least 2x genome coverage. It is shown that lcMLkin is capable for analyzing 3 to 5th degree of relatedness when 2x or higher genome coverage is available, however it is untested (in the manuscript) whether it could reliable achieve this when the coverage is less than 2x and diploid genotype likelihood assessment is questionable from WGS data. In 0.1x genome coverage samples (the limit we draw to include aDNA samples) less than 10% of genome positions and markers have sequencing coverage and almost negligible amount of sites are covered with more than 2-3 reads. On the other hand, according to our results from 2x genome coverage (and even lower c1*c2 = ~0.4 in case of haploid data) where ~99% of genome positions/markers are genotyped our method can also reliable identify 4th degree of relatives (formerly Figure 5.).

We have to note, that READ is not capable of doing such unsupervised analysis of 2136 individuals without prior knowledge of the reference population as it uses one global threshold to distinguish related people from unrelated individuals. This threshold is inferred from the analyzed data set. Furthermore, READ is technically not suitable to perform this much of samples together as one run with 2136 takes longer than six weeks on even a high end server.

To cope with this limitation, the best comparable analysis could be performed by analyzing just those populations separately with READ from this data set that have sufficient number of individuals with the same population label in our opinion. Since there are ~670+ different population labels it does not allow to do a simple meaningful comparison in most of the cases. To avoid limiting READ with the lack of proper reference population we performed the analysis only on the top 10 populations that have sufficient number of unrelated individuals usable as a reference model:

| Population | Sample size |
| --- | --- |
| Iberia_C | 52 |
| Italy_Imperial | 45 |
| Scotland_N | 37 |
| Germany_BellBeaker | 38 |
| Russia_MLBA_Sintashta | 42 |
| Czech_BellBeaker | 35 |
| Pakistan_IA_Loebanr | 37 |
| England_BellBeaker | 24 |
| Iberia_BA | 31 |
| Germany_Lech_EBA | 25 |

Our analysis (Supplementary Table 6) showed that all relatives (first and second degree) indicated by READ were also found by our methodology and the suggested degree of kinship was the same. However, our method additionally indicated one more 2^nd^ degree relatives that READ missed.

Thanks to the requested comparison we can also conclude that our analysis pipeline is several order of magnitudes quicker and scales much better for large amount of samples compared to READ software especially on multi core machines. The whole analysis took ~19 minutes for 2136 samples for the correctKin + PCangsd workflow while for 40-50 samples it took 2300+ minutes (total 7870 minutes for the top 10 populations) in case of READ (Supplementary Table 6).

We included the comparison into the Result section:

“To test the sensitivity of our analysis we used READ [26] to validate our findings. As READ depends on the proper reference population and uses a global threshold to distinguish between unrelated and potential kins the join set of 2136 individuals cannot be analyzed together. We selected the top 10 populations with the highest number of individuals and performed the READ analysis separately. READ identified relatives up to the 2nd degree. READ missed only one relation but identified no additional relatives compared to our methodology. In each cases the degree of kinship was matching between the two methods. Our method indicated one additional 2nd degree relation (AITI_95_d and AITI_98) that was missed by READ. In this case, the samples have very low genome coverage (0.145x and 0.402x) and only ~0.05% marker overlap fraction. While the corrected kinship coefficient was significantly above the 3rd degree (0.0625) relation it was less (0.1004) than the expected 0.125 corresponding to 2nd degree suggesting that these relatives share less than expected genome portions due to true biological variation (Supplementary Table 6). We had very similar scenario in case of the missed 2nd degree relation between the I1538 and I1540 CWC individuals (Table 2) suggesting that READ is less sensitive when the marker overlap is low and the shared genome fraction differs significantly from the statistically expected mean.”

Furthermore, we added the sentence “Comparison with READ showed that our method has better sensitivity, offers improved performance and scales better on multi core machines (Supplementary Table 6)” in the discussion.

Minor:

In Figure 2, do the authors know why there is a discontinuity at marker overlap fraction of 60%?

PC-Relate does need a reference population even though it does not have to be specified (by pedigree or population labels in the data set). PC-Relate does identify the matching unrelated reference by PCA. In this analysis a few EUR and EAS relatives were analyzed against AFR populations as reference. When the proper reference set is not present in the data set IBS cannot be regressed from IBD in case of relatives.

As it is an improper usage (even though non excludable usage in case of aDNA data) of this method it would be bold to suggest the true cause without knowing all the used estimators in the PC-Relate or the exact implementation of the algorithm by PCAngsd in detail.

However, PCAngsd performs a default pruning of markers below MAF 0.05. Thus based on the marker overlap different set of mainly EUR/EAS markers would be pruned when only couple of EUR, EAS individuals exist in the data set. This would lead to an analysis when the differences between EUR/EAS cannot be analyzed well as mainly the inappropriate AFR markers are kept in the analyzed data set. Since the optimal number of eigen vectors used in the analysis is calculated based on the data set it could lead to worse or better differentiation of EUR/EAS individuals depending on the marker overlap fraction. The regression of IBS against an inappropriate set of reference would be inconsistent due to this not to mention that we have very low number of true unrelated reference in this analysis. We speculate that this is the likely case of the observed anomaly.

We added the explanation in the Result section after the observation: “The results revealed that a reference population with significantly different genetic background, like African for European samples, strongly corrupts the results. In this experimental setup we lack proper number of unrelated reference hence IBS fractions are likely not represented and cannot be properly regressed out. Furthermore, when only a couple non AFR individuals are included in the analysis depending on the marker overlap fraction the EUR/EAS specific markers are mostly excluded as the PCAngsd implementation uses a default 0.05 MAF marker pruning. Thus at low marker overlap mainly the AFR specific markers are kept while at higher maker overlap slightly more EUR/EAS specific markers are also included in the analysis. Based on the used marker set the optimal number of eigen vectors and the underlying PCA based regression of IBS components are expected to be different. We speculate that these differences could be the likely cause of the observed non-linear kinship coefficient estimates in Figure 3B.”

In Figure 5, the text indicates this ranges down to about 10% marker overlap, but the plot seems to include values below 10%. Along the lines of the comments above, what percentage of marker overlap is feasible for this method to operate on? Should the plot go down to lower values?

The data and plot has as low as 1% marker overlap values as well, however we did not place proper X axe legends to display it on Figure 5. We corrected this in Figure 5. As answered earlier there is no single answer as to what marker overlap is still feasible, it is factor of the used marker set (the larger the better), the data quality and the available reference population.

Detailed comments:

- Line 16 and 44: “deeply sequenced modern DNA” and “deeply typed WGS data” - note that the samples can also be SNP array genotyped.

We included this important detail.

- Line 18-9 and 336-7: “has been possible up to the second degree with large uncertainties” and “only first and second degree relations can be detected” - but note that 1cMLkin identifies relatives of 3rd to 5th degree, so rewording this seems necessary.

In our discussion we also note:

“Based on simulated data, lcMLkin propose to accurately infer kinship up to the 3rd degree from 2x genome coverage when reference population FST is very close to analyzed data.”

In fact the first sentence of the lcMLkin paper’s discussion states: „We have demonstrated that it is possible to make accurate inference of biological relatedness down to at least three degrees of genealogical separation from 2nd generation sequencing data even when mean coverage is as low as 2x.”

However the lcMLkin is based on proper diploid genotype likelihood estimates and this method was only tested on as low as 2x genome coverage. At 2x genome coverage ~99% of markers have genotype information and likely at this coverage inferring diploid genotype likelihoods is still possible. However it is untested whether at very low 0.1x genome coverage where only ~10% of markers are covered and only haploid genotypes are feasible this method could infer proper diploid genotype likelihoods and capable to identify 3rd degree of relatives.

We smoothed our statement emphasizing “very” low coverage and changed “only” to “mainly” and also smoothed our statement on lcMLkin to mirror the result of the original manuscript:

„To date, the best analysis tools were able to indicate mainly first and second degree relatedness from very low coverage ancient samples [26, 28–30]. Based on simulated data, lcMLkin can accurately infer kinship up to the 3rd degree from 2x genome coverage when the FST is low between the reference population and analyzed data [28]. However, the majority of aDNA data is below 2x genome coverage. In these data most markers are represented by one read/genotype only. It is untested whether it is possible to infer comparable diploid genotype likelihoods suitable for lcMLkin from very low coverage data.”

- Line 38: “including GERMLINE, fastIBD, GRAB, ANGSD” - this isn’t the most recent list of methods and while that’s OK, the authors could cite Ramstetter et al. (Genetics, 2017) (which also may not be ideal since there are several even more recent methods this doesn’t consider).

As suggested we added the citation to the manuscript with the evaluation of the 12 relevant kinship analysis tools.

We tried to cite some of the established tools as an example however we agree that the list is incomplete. The number of new tools, approaches shows that this field is far from solved. We also agree that this list can never be ideal or complete since there are lots of different tools based on different principles (statistical, haplotype based, rare alleles exploited etc) that aims to work with different data (WGS, WES, array), formats (BAM, VCF, GLs) and aims to overcome different limitations (coverage, marker count, known/unknown or no reference population at all).

- Lines 45-46: “an appropriate set of reference data” - note that not all algorithms explicitly (or even implicitly) use a reference set. KING and IBIS, for example, only analyze the genotypes of each sample pair to quantify their relatedness.

Thank you for the correction, we included this extra information in the manuscript.

- Although the analyzes as given may give an adequate picture, it would be better to do more than three simulations (Table 1). Doing several dozen would allow the authors give a mean and standard deviation (for example).

As stated in a previous answer we performed additional simulations analysis to assess mean and SD of estimated kinship coefficients between different randomizations (haploid state and marker subset) at different marker overlap fractions. We included the results in Supplementary Table 1.

- To help readers, it would be helpful if the number of SNPs included in each analysis was given in the caption. That is, for analyzes where no down sampling (or down sampling to 1240k), please state this in the captions (e.g., Table 1).

Since we used modern and ancient data it is not obvious reading the Results that in all of our analyses (including modern data) were performed/down sampled data to the 1240K data set.

Even though we stated this information in the Methods:

# *“Methods*

## Used data sets

*In all of our analysis we used the genome coordinates of 1240K SNP set from Allen Ancient DNA Resource [23]. For marker overlap simulations we used two different full typed modern data sets: the 1000 Genomes Project Phase 3 data [25]; and a large admixed Cabo Verdean-Hungarian family of known pedigree with first (siblings), second (half siblings, and fifth degree relatives) from our anonymized clinical biobank. The variants of the joint VCF (Variant Call Format) files were filtered for the 1240K SNP coordinates and imported into plink 1.9 binary format [32].”*

Due to the manuscript structure the reader only gets to this important information at the end, thus we also stated this in the beginning of the Result section:

“To use marker counts similar to aDNA data, all modern dataset were down sampled to the autosomal marker positions of the 1240K SNP set of the AADR dataset [23] in all of our simulations.”

- Line 244-5: “represents 6 sigma threshold” - is this for unrelated pairs?

Indeed, we presume that majority of the pairwise relations in the data set are unrelated, and the 6 sigma threshold was calculated compared to the statistics of the estimated kinship coefficients. The rationale is that when we analyze the test individuals with a reference population then true kin relations are only extremely low fraction of all unrelated combinations between the analyzed individuals.

We have to note that the 6 sigma threshold is subjective, we used this as smartpca uses this threshold for identifying „biological” outliers. Accordingly, above 6 sigma threshold means that it is very high chance that the higher kinship coefficient represents biological difference (either IBD of real relatives, or some minor component in a subset of samples that is missing from the reference population) and is not a technical error of the method.

- Line 256-7: “the analysis shows that the correction itself does not significantly multiply the error rate” -- this may be a bit too strong. Perhaps including more pairs and giving standard errors for the kinship coefficients of pairs with known relatedness would make this statement easier to back up with data.

In our new simulations we addressed this problem and the increase in SD is significantly smaller than the applied correction factor (4x vs 20x at 0.05 marker overlap fraction), thus we assume that the increased SD is mainly not because of the correction but due to higher uncertainty of the PCA analysis because of the smaller number of markers used.

We noted this in the results and smoothed the meaning of our sentence: “It is expected that individuals genotyped at low partially overlapping subset of markers would lead to less complete representation of the reference population and the test individuals thus regression of IBD/IBS based on the PC-Relate algorithm would have higher SD at partially genotyped data. Although the marker overlap fraction correction differs more than one magnitude between very low and high marker overlap fraction sample pairs, the analysis shows (Supplementary Table 1) that the correction itself does not significantly multiply the error rate (4x increase in SD at 20x correction factor) and the increased SD of the method are likely due to the higher uncertainty of PCA.”

However, considering Figure 5, and that the correction factor is slightly more than 2 orders of magnitudes higher for very low marker overlap (0.007) and almost fully typed (0.996) fractions while the SD of corrected kinship coefficient of unrelated kinship coefficient only increases like 4 fold only we can safely state that the increased uncertainty of the corrected kinship coefficient is likely due to the higher uncertainty of PCA regression from lower amount of markers. We described this in the results section: “Although the marker overlap fraction correction differs two magnitudes (0.007- 0.996) between very low and high marker overlap fraction sample pairs, the analysis shows that similar to the corrected kinship coefficient between relatives (Supplementary Table 1) the correction itself does not significantly multiply the error rate of unrelated samples. Most of the technical errors are expected to be the result of using very sparse data to regress out IBS to identify IBD fragments by PC-Relate.”

- Line 304-5: “we filtered potential relatives above 0.046875 corrected kinship coefficient” - what is the reason for this filter?

As stated before, this analysis setup in the REICH dataset is far from ideal. We analyzed together a large set of individuals with largely different genome coverages (0.048x-25.372x) from 673 different populations of various genome structures. In this setup we have no knowledge which individual had proper and/or sufficient number of surrogate populations. According to our results missing or bad reference population leads to inflated false positive kinship coefficient estimation. Since at this analysis PC-Relate identified 7 eigen vectors as optimum it also shows that we were stretching the limits of the methodology for identifying very distant relatives as analyzing this much populations together would lead to miss the fine population structure of some analyzed individuals and populations. Thus many of the presumed hypothesizes were shaky in this analysis. To avoid identifying false positive relative groups we used a stricter criterion for this specific analysis only. In our software we have option to filter relatives based on sigma or kinship coefficient, we chose the latter and the theoretical lower limit (0.046875) of 3rd degree of relatedness was applied.

- Line 353: “lead to major bias” - this could be right. Are there any papers that show this? If not, perhaps being a little less certain in the wording (and explaining the reasoning) would be good.

We had not read this explicitly in the manuscripts that we know of hence we changed the sentence to less certain: „…could lead to major bias”.

- Line 372: “as used in prior protocols” - perhaps citing here would help?

In a review about kinship analysis of ancient samples I’ve read that classifying samples by their coverage and analyzing same coverage samples together could potentially help the variable genome coverage problem. But I’ve read so many manuscripts, that unfortunately I cannot recall and find the one.

Due to the changes suggested by the other reviewer, this paragraph was removed as it was closely resembling to a paragraph in the Results/*The effect of overlapping marker fraction on the kinship coefficient calculation* section (lines 149-153) where this bold statement is missing.

- Line 412: “our approach could properly identify all relations up to 4th degree” - but how do we know what the true relationship is here?

In this case in the original READ manuscript the 3rd and 4th relations can be inferred from the „chain” of multiple first and second degree relations of this large group of relatives (see figure 6 in the manuscript: <https://pubmed.ncbi.nlm.nih.gov/29684051/#&gid=article-figures&pid=fig-6-uid-5>). Our analysis directly identified these indirectly inferred 3rd and 4th relations as the corrected kinship coefficients matched the expected kinship coefficient.

- Figure 6: I wasn’t totally sure I knew what happens at each stage of this workflow. For example, is PCA calculated by PCAngsd? Adding to the caption and to the Methods (or other) sections would help specify the way the approach works. On this figure, what is the difference between the curated PLINK dataset and fully typed/modern samples?

Perhaps the figure was not descriptive enough, at the first few steps we import and shape data from various data sources. So that we end up in the required PLINK or EIGENSTRAT data format. The PCA is the integral part of PCAngsd kinship coefficient estimation, that implements the PC-Relate algorithm and works with PLINK or EIGENSTRAT data.

The main difference between usable data sources are usually due to the used state-of-the-art tools and their different data format. In case the BAM files are available then ANGSD can be used to random allele calling however we need to convert the output of ANGSD into PLINK. In case of modern data it is rare that data is available in the restricted biallelic PLINK/EIGENSTRAT format. In this case usually the joint VCF of samples is available and this data can be imported by PLINK. In case we have already a curated PLINK data set like the REICH data set it can be used after random pseudo haploidisation.

We remade the figure to differentiate the data shape/import part from the actual analysis and model based kin filtration to avoid confusion.

Reviewer #2: Nyerki et al describe some observations about the performance of PC-relate with regard to low-coverage data. They use the knowledge from these observations to implement a normalization scheme that greatly increases the sensitivity of this program for detecting more distant relations. The design and description of their work is adequate. Helpfully, they analyze the large REICH dataset, detecting many instances of apparent sample mislabeling.

This is a nicely conceived and executed piece of work. I have several suggestions that the authors may mind helpful, listed below.

1. The central observation is that kinship coefficient calculated by pcrelate is strongly, linearly correlated with the number of markers available in the comparison. This observation is demonstrated very clearly. This observation, however, may simply be described as a bug in pcrelate. It seems as though the authors of pcrelate may not have contemplated the case of an incomplete dataset and simply do not divide some number by the appropriate denominator. In any case, once this result is shown (Figure 1), it does not add much to the paper to include the "marker overlap fraction" to all subsequent figures. The reader will get this simple point from the authors' clear explanation and the results of figure 1. Consider simplifying the subsequent figures in this way.

The PC-Relate algorithm was developed, tested on diploid modern data thus the original manuscript never intended to deal with the possibility of using incomplete, low genotype certainity or haploid data sets associated with aDNA studies. We agree with the reviewer that this lack of correction in the original PC-Relate algorithm for marker counts is a shortfall of the original implementation, however we would not call it a „bug” as these scenarios were never been considered and tested in the original PC-Relate manuscript. We added the sentence „The original PC-Relate algorithm was created to analyze modern fully genotyped diploid samples. However, in the case of ancient data, genome coverage and partial genotyping is one of the factor that has the greatest variability between samples.” in the Results/*The effect of overlapping marker fraction on the kinship coefficient calculation* section to clearly state this methodological shortfall to the readers as requested in point 7. also.

Since the marker overlap fraction (or rather the number of data points used for the analysis) does alter the probabilities and the standard deviation of the kinship coefficient estimation, while theoretically we see a strong linear correlation still the statistical reproducibility and power of the analysis (standard deviation of the whole method depending on the marker overlap fraction) highly depends on this factor. As it is shown in our new simulations (Suplementary Table 1). Thus as it is also shown in figure 5. we think this information shows this relation to the reader

Considering all reviewer’s feedback, we restructured the Figures to demonstrate the key points and Figures in the main manuscript, while putting all the important but repetitive information into the Supplementary data.

2. The figures, in general, are not well labeled. The authors rely on putting information in the figure captions. A well-designed figure does not require much of a caption as all the labeling is in the figure itself. In particular, Figure 2 should have the 1000Genomes population labels included in the legend of the figures. For example: GBR (HG00124-HG00119; 2nd degree)). Also, in Figure 3, the axis labels in Panel a and b are identical. One must read the caption to learn how they differ. Why not put that information in the figure itself?

We added the requested information in figure captions and also applied the same principle at our newly added two figures.

3. The analysis of the mixed ethnicity Hungarian cohort showed that using allele frequencies from multiple panels "fixed" the problem of using the wrong population. This suggests that a better approach for some (maybe all?) samples would be to use a more general population model. The authors should test their method using all of 1000Genomes as the "population". They may find that the wonky behavior of using the "wrong" population is fixed by simply using a general, comprehensive model of "human" allele frequencies as the background PCA model.

As the reviewer suggests this „all in” population data set used as reference could be a quick automated approach for most of the cases. However, we have to note that in practice, PC-Relate algorithm tries to guess the optimal number of eigen vectors for the PCA analysis in the first step based on the analysis of the whole data set. Thus when many different populations are present the differences on the main PC components could be much bigger than for example the differences between the genome composition in our test individuals. For example the fine genome structure of European populations would not be as precisely represented as it could be when omitting widely different populations (for example African) from the analysis that have no genome components in our test individuals. Thus as we had noted the best case is still when the appropriate reference populations are present. However, this would require either prior knowledge, a larger sample set from the analyzed individuals or additional analyzes (like Fst or fastNGSAdmix) to find unrelated individuals in an objective way that largely have similar genome structure compared to our test individuals.

We noted this in the discussion: „When no prior knowledge exists on the reference population F_ST_ or FastNGSAdmix [32] analysis could be used as an objective method to select individuals best matching our test individual’s genome structure as a reference population.”

4. Line 17, "population" should be "populations"

We corrected the sentence.

5. The second paragraph of the background alternates between a general description of the problem to specific minutiae of their implementation (using only bi-allelic, high minor-allele frequency SNPs). This section could be better written with shorter, more coherent paragraphs.

As suggested we restructured the Background, we kept the second paragraph to detail the problem and moved the proposed solution to the next paragraph where we described our aims.

6. Line 116 "where 50-50%" is unclear

We rewrote the sentence to be more clear.

7. The sentence in Lines 133-135 seems to be the central "normalization" done to fix what might be described as a bug in pcrelate. As written, this seems to be a minor tweek. In fact, it's the main insight! As written, the reader may not appreciate how simple the solution is. Consider contextualizing this statement appropriately.

As suggested we tried to better contextualize our major finding at this section:

„The original PC-Relate algorithm was created to analyze modern fully genotyped diploid samples. However, in the case of ancient data, genome coverage and partial genotyping is one of the factor that has the greatest variability between samples. Kinship coefficient calculation is based on the IBD segments shared between two samples which can only be assessed at marker positions where both samples are genotyped. To investigate the effect of this factor we defined a metric called overlapping marker fraction. We calculate this metric by dividing the number of markers where both samples are genotyped with the total number of markers in the dataset (1240K).”

8. Figure 2, panel b has an interesting anomaly around marker overlap fraction 60. The authors may want to speculate why the trend of kinship coefficient is discontinuous through this region of marker overlap fraction.

PC-Relate does need a reference population even though it does not have to be explicitly specified (by pedigree or population labels in the data set). PC-Relate does identify the matching unrelated reference by PCA. In this analysis a few EUR and EAS relatives were analyzed against AFR populations as reference. When the proper reference set is not present in the data set IBS cannot be regressed from IBD in case of relatives.

As it is an improper usage (even though not fully excludable scenario in case of aDNA data) of this method it would be bold to suggest the true cause without knowing all the used estimators in the PC-Relate or the exact implementation of the algorithm by PCAngsd in detail.

However PCAngsd performs a default pruning of markers below MAF 0.05. Thus based on the marker overlap different set of mainly EUR/EAS markers would be pruned when only couple of EUR, EAS individuals exist in the data set. This would lead to an analysis when the differences between EUR/EAS cannot be analyzed well as mainly the inappropriate AFR markers are kept in the analyzed data set. Since the optimal number of eigen vectors used in the analysis is calculated based on the data set it could lead to worse or better differentiation of EUR/EAS individuals depending on the marker overlap fraction. The regression of IBS against an inappropriate set of reference would be inconsistent due to this not to mention that we have very low number of true unrelated reference in this analysis. We speculate that this is the likely case of the observed anomaly.

We added the explanation in the Result section after the observation: “The results revealed that a reference population with significantly different genetic background, like African for European samples, strongly corrupts the results. In this experimental setup we lack proper number of unrelated reference hence IBS fractions are likely not represented and cannot be properly regressed out. Furthermore, when only a couple non AFR individuals are included in the analysis depending on the marker overlap fraction the EUR/EAS specific markers are mostly excluded as the PCAngsd implementation uses a default 0.05 MAF marker pruning. Thus at low marker overlap mainly the AFR specific markers are kept while at higher maker overlap slightly more EUR/EAS specific markers are also included in the analysis. Based on the used marker set the optimal number of eigen vectors and the underlying PCA based regression of IBS components are expected to be different. We speculate that these differences could be the likely cause of the observed non-linear kinship coefficient estimates in Figure 3B.”

9. In figure 5, there are many sample comparisons that appear to be 1st, 2nd and 3rd degree relatives. Are these known relationships? That is, should these be considered positive controls or errors? In either case, they should be labeled and discussed.

In the original 1KG data related individuals are indicated up to second degree of relations. We identified the exact same first and second degree relations by our method between the analyzed subset (the used subset of EUR, EAS individuals) indicated in the 1KG pedigree data thus this could be regarded as positive controls. As there was no pedigree data collected from 1KG individuals other than first and second degree of relations the indicated third and fourth level of relatedness is outcome of our current method. In supplementary Table 3 we compared the estimated kinship coefficients of indicated relatives between the full diploid, full haploid, depleted diploid and depleted haploid data set.

We included the original pedigree information of the 1KG pedigree data to Supplementary Table 4 and included the following sentence in the manuscript:

“Our method identified all known 1^st^ and 2^nd^ degree relatives that were included in the analyzed subset of 1KG EUR/EAS individuals and indicated a couple of additional distant 3^rd^ or 4^th^ relatives (Supplementary Table 4).”

10. Line 335, the phrase "Fst is very close…" should be changed to "Fst is very low"

We changed the sentence.

11. The authors may care to comment on the following suggestion: Perhaps their method would be more robust to population knowledge if the first pre-selected only markers that have low Fst between populations. In this way, it may not be necessary to know, a priori, what the appropriate background model should be.

It is a very interesting idea to only use markers that have very similar allele frequencies hence leading to low FST between populations. However, this criterion would most likely severely restrict the number of usable markers. Also in my opinion more experimental data and analysis should be performed to ensure that this approach could be used in case of small drifting populations (often the case for ancient groups) or admixed people. Although a different solution to the problem, when no prior knowledge exists on the proper reference FST or FastNGSAdmix analysis could likely yield appropriate reference individuals from the ever growing ancient data base.

Since the other reviewer was also concerned about how to solve the missing (population label) or unknown reference issue in case of aDNA we added the sentence: „ When no prior knowledge exists on the reference population F_ST_ or FastNGSAdmix [32] analysis could be used as an objective method to select individuals best matching our test individual’s genome structure as a reference population.” to the Discussion to address this concern.

12. Line 434 "…already fixated at this age". I assume the authors mean "…already fixed at this age", i.e., were at 100% population frequency and not segregating variation. In any case, that is unlikely to be true since fixed sites should be less informative in this analysis.

We likely used the wrong terminology coming from maternal and paternal haplogroup data (ie marker fixation age). We rephrased this sentence to: „On the other hand, our results show that the 1240K marker set was sufficient to properly analyze 4000 year old ancient Corded Ware Culture individuals with a modern Eurasian reference population, suggesting that the majority of the high frequency EUR informative markers were already present at this age.”

13. In general, the discussion is a bit tedious. It is mostly a rote enumeration of the results. It should be shortened, focusing on contextualizing the results.

To address this suggestion, we moved the most obvious findings and interpretations to the Result section and tried to simplify the Discussion to avoid unnecessary repetition from the Background and Results.

14. The authors are to be commended for their efforts to detect and describe sample mix-ups in the Reich dataset.

We deeply appreciate all the constructive criticism of the reviewer!

**Second round of review**

**Reviewer 1**

The authors did a fine job addressing many of my prior concerns. As a suggestion, it would be nice to characterize the performance of the method using better surrogates for aDNA samples – particularly using samples that are more inbred or drifted compared to the modern samples used for most of the validation. Simulated data could be used for this purpose. However, it’s clear that the methods have utility in many settings and therefore I’m supportive of publishing this work.

A few other questions/comments the authors may wish to address:

- In Figure 1B, the points are not centered at the expected values for sample duplicates and 1st and 2nd degree relatives. Does this indicate that the method has a downward bias overall? What is the source of these reduced kinship coefficients?

- I may have missed it, but it wasn’t obvious how the estimated kinship coefficients get mapped to a degree of relatedness call. Also, does the marker overlap fraction influence whether a sample is called as related or not? The red bands in Figure 5 suggest the need to do so. Here clarifying the text on lines 387-390 might be helpful.

- Along the lines of the above, while the plots convey estimated kinship coefficients, what would be nice to see – particularly in the context of Figure 2 and the real data analysis – is how the altered genotypes affect the precision and recall of degrees of relatedness (or sensitivity/specificity). I know the truth in the real data is generally unknown, but some high level quantification of performance would be helpful. (See also lines 247-8: “thus the estimated degree of relatedness remained the same” – how much of a drop/what kinship value would lead to a different degree of relatedness?)

- Lines 374-9 give the correlation between the 1.2M diploid data and the reduced coverage kinship coefficients. Are there overall biases, positive or negative when one reduces coverage?

- Lines 457-8 say “READ missed only one relation”. Is that the pair described two sentences later (“Our method indicated one additional 2nd degree relation”)? I’m confused because a little later the text reads “We had a very similar scenario in case of the missed 2nd degree relation”. Are there two 2nd degree pairs READ missed?

Minor:

- Line 141: shouldn’t say “exactly 50%”, but rather 50% on average or something (also see line 145).

- Line 146-7: “using their own reference population for the 100 different randomizations” – my first read of this made me wonder if the reference sample was somehow used for the randomization. I assume what is meant is that the PCA used the same reference population as the sample and that 100 random pseudo-haploidizations were done?

- Line 173: “dups” -> “duplicates”.

- Line 423: “from only the family relations” – the response to reviewers described this more clearly. Might be nice to make more explicit the fact that the inferred 1st (and 2nd) degree relationships were used to obtain the more distant pairwise relationships.

- Pg 35 and 40 have placeholders XXXX and ZZZZ.

- At least one supplementary Figure references the REICH dataset instead of the current AADR.

**Reviewer 2**

The authors have adequately addressed my issues with this manuscript.

**Authors Response**

**Point-by-point responses to the reviewers’ comments:**

The authors did a fine job addressing many of my prior concerns. As a suggestion, it would be nice to characterize the performance of the method using better surrogates for aDNA samples – particularly using samples that are more inbred or drifted compared to the modern samples used for most of the validation. Simulated data could be used for this purpose. However, it’s clear that the methods have utility in many settings and therefore I’m supportive of publishing this work.

A few other questions/comments the authors may wish to address:

- In Figure 1B, the points are not centered at the expected values for sample duplicates and 1st and 2nd degree relatives. Does this indicate that the method has a downward bias overall? What is the source of these reduced kinship coefficients?

*Response: The reviewer is right that the method has a slight downward bias in case of the analyzed 1KG sample duplicates (n=5, mean kin. coeff. = 0.4935; ~1.5%) and 1st degree relatives (n=2, mean kin.coeff. = 0.2389, ~4.4%). This can also be noticed in case of the validated archaic sample duplicates (n=20, mean kin. coeff. = 0.4801, ~4%) and 1st degree relatives (n=119, mean kin. coeff. = 0.2405, ~4%). In the case of 2nd degree or more distant relatives the proportion of shared IBD has true biological variation due to segregation and recombination. In fact the selected individuals with 2nd degree relations in Figure 1B have higher than average shared IBD fragment proportion. This is likely due to true biological difference and higher than average IBD fragment proportion shared between these relatives. Diploid fully typed data generates the same coefficient. Since individual deviations from the shared genome portions are random the mean of the corrected kinship coefficient of a statistically large cohort of relatives should be close to the predicted value when there is no overall bias. In case of the archaic AADR data the mean of the corrected kinship coefficient of the indicated 2nd degree relatives (n=119) is 0.1274 that is a slightly over the expected value (~2% relative difference). This mean that the observed deviations are likely a result of more than a single factor.*

*There are several estimators in the PC-Relate algorithm for allele frequencies, population shared and individual shared proportions of the genome. The experimental bias and the interaction between these factors in case of simulated and/or archaic data is not known. Uncovering the source of bias would require much deeper understanding of the math and an extensive analysis of all of the underlying estimators using experimental data.*

*We do agree that our method could be further improved however the observed 1-2% relative deviations do not significantly affect the outcome of the kinship estimation and we think that in depth investigating the underlying cause is beyond the scope of this manuscript.*

- I may have missed it, but it wasn’t obvious how the estimated kinship coefficients get mapped to a degree of relatedness call. Also, does the marker overlap fraction influence whether a sample is called as related or not? The red bands in Figure 5 suggest the need to do so. Here clarifying the text on lines 387-390 might be helpful.

*Response: Indeed our relative filtration is based on the observed experimental variance of the corrected kinship coefficient across different marker overlap fractions. Thus we have different 6 sigma thresholds to distinguish between unrelated and related individuals that is a factor of the marker overlap fraction and is based on the actual variance in the dataset (as shown in Figure 5). In the current state of the software the corrected kinship coefficient is mapped to the most likely degree of relatedness only. However, it provides all statistical evidence and limitations to help assess this “judgement”. We do see that it would be more user friendly to not only output the corrected kinship coefficient, the 95% confidence interval (that is based on the variance observed at the given marker overlap fraction) and the 6 sigma threshold, but also provide interpretation of the data such as “consistent with …, but not excluding …”. Due to the journal policy we are required to deposit our software as used in the manuscript. We expect to implement suggestions, improvements based on community feedback on our github repository of correctKin. Accordingly we clarified this point in the manuscript: “Based on the observed variation at different overlap fractions we can statistically differentiate unrelated and related pairs of individuals. Since in this experiment we couldn’t exclude errors due to missing genome components from the reference populations, we used a conservative 6 sigma threshold to identify biological differences. As seen in Figure 5, this statistical threshold depends on the fraction of marker overlap and the quality of the data (diploid/pseudo-haploid).”*

- Along the lines of the above, while the plots convey estimated kinship coefficients, what would be nice to see – particularly in the context of Figure 2 and the real data analysis – is how the altered genotypes affect the precision and recall of degrees of relatedness (or sensitivity/specificity). I know the truth in the real data is generally unknown, but some high level quantification of performance would be helpful. (See also lines 247-8: “thus the estimated degree of relatedness remained the same” – how much of a drop/what kinship value would lead to a different degree of relatedness?)

*Response: The absolute difference of the expected kinship coefficient between consecutive degree of relatives is halved (0.5, 0.25, 0.125, 0.0625, …) with each additional degree of relatedness. Accordingly, a 50% relative drop in the estimated kinship coefficient would be equal to the next degree of relation. This is in fact exactly stated in the manuscript in the beginning of the sentence “In our simulations even at the applied relatively high error rates (compared to experimental aDNA error rate) the largest effect was still significantly smaller than the 50% difference of expected kinship coefficients between different degrees of relations thus the estimated degree of relatedness remained the same.”*

*As seen in Figure 2, even at double of the critical/FAIL contamination threshold the relative drop in kinship coefficient is significantly smaller than the difference between two degrees of relatedness. It is likely that a correction considering this factor could be used, however it would make automation of the analysis much harder as we would need extra information that is not contained in genomic datasets and is not always available. Furthermore, it is not trivial how different combinations of the type and level of contamination in the related sample pair would alter the outcome. In the Discussion we addressed this issue and noted that typical aDNA contamination (based on the AADR dataset) does not significantly alter the results and highly contaminated samples could invalidate the results:*

*“The mean corrected kinship coefficient of the validated sample duplicates and 1st degree relatives of the experimental 2136 AADR individual was 0.48 and 0.24 respectively (approximately ~4% lower from the expected). This is in accordance with the mean X-contamination rate (1.28%) of ancient individuals of the AADR V42.2 dataset suggesting that our kinship-estimation method can be safely used on typical aDNA data. Nevertheless, analysis of highly contaminated (CRITICAL/FAIL) samples containing higher rate of genotyping errors (>5%) could lead to underestimation of corrected kinship coefficient and as a result to underestimation of the degree of relation especially in case when the relatives share less than the expected IBD fragments due to true biological variation.”*

- Lines 374-9 give the correlation between the 1.2M diploid data and the reduced coverage kinship coefficients. Are there overall biases, positive or negative when one reduces coverage?

*Response: Our results suggest that the correlation is strongly linear thus coverage as a factor and the applied correction is not the source of the positive or negative bias. To be precise we have to note that at extremely low marker count this linear correlation was not tested and as mentioned earlier some of the PC-Relate estimators could be based on a hypothesis that is violated at very sparse datasets. We stated in the manuscript that extremely low marker count (0.1x or lower) genome coverage over sequenced samples tend to introduce increased amount of genotyping bias and such samples are rightfully excluded from most population genetics analyses.*

- Lines 457-8 say “READ missed only one relation”. Is that the pair described two sentences later (“Our method indicated one additional 2nd degree relation”)? I’m confused because a little later the text reads “We had a very similar scenario in case of the missed 2nd degree relation”. Are there two 2nd degree pairs READ missed?

*Response: At lines 457-8 we discuss our finding of the CWC relatives analyzed and compared with the result of the original READS manuscript. At the next paragraph we present our findings on the unsupervised analysis of the 2136 ancient individuals from the AADR data set. As previously requested we performed a validation of our findings with READ using the top 10 populations where we had sufficient amount of population matched samples as reference to run READ. Both methods resulted in the same relatives and degree of relations, however at this comparison our method indicated one additional 2nd degree relative that was missed by READ as shown in Supplementary Table 6. In both cases of the missing 2nd relatives, the slightly lower than expected proportion of IBD fragments appeared to be the cause.*

Minor:

- Line 141: shouldn’t say “exactly 50%”, but rather 50% on average or something (also see line 145).

*Response: In case of parent offspring, with very rare exception due to de novo structural variants, 50% of genome is inherited from one parent. 2nd degree or more distant relations statistically share 25% of their genome with their grand relatives, due to uneven recombination and consecutive segregation. We wanted to distinguish between these two scenarios, however we have removed the word “exactly” from the manuscript.*

- Line 146-7: “using their own reference population for the 100 different randomizations” – my first read of this made me wonder if the reference sample was somehow used for the randomization. I assume what is meant is that the PCA used the same reference population as the sample and that 100 random pseudo-haploidizations were done?

*Response: Thank you for your right observation. We rephrased the section as: “We calculated the kinship coefficient for each of the selected relatives (kin1/kin2) for the 100 different randomization using their own reference population and calculated the mean and the standard deviation of the estimated kinship coefficients.”*

- Line 173: “dups” -> “duplicates”.

*Response: We corrected all occurrences in the manuscript.*

- Line 423: “from only the family relations” – the response to reviewers described this more clearly. Might be nice to make more explicit the fact that the inferred 1st (and 2nd) degree relationships were used to obtain the more distant pairwise relationships.

*Response: We rephrased the sentence: „These individuals were analyzed in the original READ manuscript where relations were identified up to the 2nd degree except the missed 2nd relation between I1538 and I1540 while the more distant 3rd and 4th relations were only indirectly indicated based on the inferred 1st and 2nd relations.”*

- Pg 35 and 40 have placeholders XXXX and ZZZZ.

*Response: We added the proper references to our data sets and software*

- At least one supplementary Figure references the REICH dataset instead of the current AADR.

*Response: We had checked and corrected all references to refer the AADR data set.*

**Thirds round of review**

**Reviewer 1**

I thank the authors for the response. The manuscript hasn't changed much in response to my prior comments, and a few additions/clarifications are important to include. More specifically:

- A comment noting the downward bias and the other responses given regarding second degree relatives would be helpful.

- To ensure that the manuscript has sufficient information, please provide details on how the software maps kinship coefficients to degree. What function is used? How is that function fit to the data?

- It's surprising to me that the altered genotypes in Figure 2 never produce a different degree of relatedness call. Is the same likelihood function as above used to calculate kinship coefficients here? It's not fair to simply check that the kinship coefficient drops by less than 50% of the relative differences between the degrees of relatedness. For example, a pair with kinship coefficients of 0.18 is likely second degree related, but if their kinship coefficient drops to 0.16 and if we used the bounds KING uses to map kinship coefficient to degree, the pair would be called as third degree relatives. I'm confused here and hoping that the original degree isn't used to decide what degree should be mapped to under the altered kinship coefficient.

- I didn't see a full answer to the question regarding the correlation between the 1.2M diploid data and the reduced coverage kinship coefficients. Are there overall biases, positive or negative when one reduces coverage?

- Thank you for clarifying the text regarding READ missing only one relation, etc. This is worth adding to the paper as others may similarly be confused by the current wording.

**Authors Response**

**Point-by-point responses to the reviewers’ comments:**

I thank the authors for the response. The manuscript hasn't changed much in response to my prior comments, and a few additions/clarifications are important to include. More specifically:

*Response: Dear Reviewer, thank you very much for the clear explanation of the raised points. Please find our detailed answers and the modifications we made in the manuscript below.*

- A comment noting the downward bias and the other responses given regarding second degree relatives would be helpful.

*Response: We added a note concerning the overall biases we observed on the experimental data in the Discussion: “According to our results, the used method had slight downward (2-4%) bias in the analyzed 1KG dups and first degree relatives and also in the validated first degree ancient samples. However, this downward bias is also present in the kinship coefficient estimation of fully typed diploid 1KG relatives suggesting that the original PC-Relate algorithm and not the applied correction or pseudo-haploidization is accountable for this bias. This is also supported by our simulations on the corrected kinship coefficient calculated from marker depleted pseudo-haploid data and the original fully typed diploid data (Additional File 2: Table S1, Figure 1B) and the very high correlation between the kinship coefficient calculation of marker depleted pseudo-haploidized and the original fully typed 1KG data (Additional File 5: Table S4). On the other hand, the mean of the corrected kinship coefficient of the indicated 2nd degree relatives (n=119) of experimental AADR data is 0.1274 (Additional File 6: Table S5) that is a slightly over the expected value (~2% relative difference) suggesting that the bias could originate from more than a single factor.”*

- To ensure that the manuscript has sufficient information, please provide details on how the software maps kinship coefficients to degree. What function is used? How is that function fit to the data?

*Response: We use asymmetric kinship coefficient thresholds. The lower bound is the mean of the kinship coefficients corresponding to the given degree and one-degree higher relation, while the upper bound is the mean of expected kinship coefficients corresponding to the given degree and one-degree lower relation. Accordingly, we used the [0.1875, 0.375], [0.09375, 0.1875], [0.046875, 0.09375] and [0.0234375, 0.046875] bounds as thresholds to map 1st, 2nd, 3rd and 4th relations. This is slightly different compared to the thresholds [0.177, 0.354], [0.0884, 0.177] and [0.0442, 0.0884] used for 1st, 2nd and 3rd relatives at KING. The most likely degree of relation is mapped using these thresholds to evaluate the calculated corrected kinship coefficient. We added this information in Additional File 8: Supplemental Note; Method description at the section Filtering the corrected kinship coefficient matrix for relatives.*

- It's surprising to me that the altered genotypes in Figure 2 never produce a different degree of relatedness call. Is the same likelihood function as above used to calculate kinship coefficients here? It's not fair to simply check that the kinship coefficient drops by less than 50% of the relative differences between the degrees of relatedness. For example, a pair with kinship coefficients of 0.18 is likely second degree related, but if their kinship coefficient drops to 0.16 and if we used the bounds KING uses to map kinship coefficient to degree, the pair would be called as third degree relatives. I'm confused here and hoping that the original degree isn't used to decide what degree should be mapped to under the altered kinship coefficient.

*Response: In the examples of the manuscript it did not cause any changes in the classification, please see the attached excel sheet containing the raw data of the corrected kinship coefficient calculations from the 100 different genotype error randomization for the 4 different genotype error scenarios presented in Figure 2. In all cases we got the same classification. Please also note, that we displayed the 95% confidence interval and not the SD at Figure 2 (and also at Figure 1) while in the KING manuscript the SD is displayed (https://www.ncbi.nlm.nih.gov/pmc/articles/PMC3025716/figure/F1/).*

*As the reviewer rightly suggests, since in siblings, second and higher degree of relations there is true biological variation and in case of much lower IBD is shared from the statistically expected value between two relatives (due to skewed recombination and segregation) and high amount of contamination or genotyping errors could lead to falsely identify such individuals with higher degree of relation. To better interpret the results, we modified our conclusion at this section: “In our simulations even at the applied relatively high error rates (compared to experimental aDNA error rate) the largest effect was still significantly smaller than the 50% difference of expected kinship coefficients between different degrees of relations thus the estimated degree of relatedness of the analyzed relatives remained the same. However, we have to note that skewed IBD sharing and high genotyping error in the test individuals could lead to false (one-degree higher) classification of the analyzed relation.”*

In the Discussion we already noted, that misclassification could happen due to highly contaminated samples in this scenario: “Genotyping error simulations show that approximately double error rate compared to typical experimental aDNA data leads to 5.4-10.4% proportionally lower corrected kinship coefficient than the expected kinship coefficient in our workflow (Figure 2, Additional File 3: Table S2). The mean corrected kinship coefficient of the validated sample dups and 1st relatives of the experimental 2136 AADR individual was 0.48 and 0.24 respectively (approximately ~4% lower from the expected). This is in accordance with the mean X-contamination rate (1.28%) of ancient individuals of the AADR V42.2 dataset suggesting that our kinship-estimation method can be safely used on typical aDNA data. Nevertheless, analysis of highly contaminated (CRITICAL/FAIL) samples containing higher rate of genotyping errors (>5%) could lead to underestimation of corrected kinship coefficient and as a result to underestimation of the degree of relation especially in case when the relatives share less than the expected IBD fragments due to true biological variation.”

*Furthermore the original degree is not used to decide what degree of relation should be mapped. As stated in the manuscript and also at the detailed description in Additional File 8: Supplemental Method; Filtering the kinship coefficient matrix for relatives: we used the 6 sigma threshold (by default) of the corrected kinship coefficient to differentiate unrelated and related individuals, thus the variation in the experimental data is used to decide which corrected kinship coefficient threshold is sufficient to call as a relative and mapped to the most likely degree of relation or not. Our analysis shows that due to the very low SD of the methodology this threshold is much lower than the fix 0.0884 threshold used in KING to identify family relatives.*

*We added the following sentences to the Result section to explicitly state this: Statistical validation, assessment of technical errors “The variance of the corrected kinship coefficient depends on the marker overlap fraction between the test individuals (Figure5.). Since the marker overlap fractions between any two ancient samples are different applying a pre-defined kinship coefficient threshold to identify relatives would lead to decreased sensitivity or specificity depending on the marker overlap fraction. In other words the statistical power to differentiate relatives from unrelated depends on the marker overlap fraction and the same threshold shouldn't be applied. However, based on the experimental variance of the corrected kinship coefficient observed in the analyzed dataset the Z score could be used as a criteria to differentiate relatives from unrelated with the same sensitivity and specificity independent of the marker overlap fraction.”*

- I didn't see a full answer to the question regarding the correlation between the 1.2M diploid data and the reduced coverage kinship coefficients. Are there overall biases, positive or negative when one reduces coverage?

*Response: (- Lines 374-9 give the correlation between the 1.2M diploid data and the reduced coverage kinship coefficients. Are there overall biases, positive or negative when one reduces coverage?)*

*The question refers to these lines in the manuscript (underlined lines 374-379 /in the previous revision/ with the context):*

*„Although the marker overlap fraction correction differs two magnitudes (0.007- 0.996) between very low and high marker overlap fraction sample pairs, the analysis shows that similar to the corrected kinship coefficient between relatives (Additional File 2: Table S1) the correction itself does not significantly multiply the error rate of unrelated samples. Most of the technical errors are expected to be the result of using very sparse data to regress out IBS to identify IBD fragments by PC-Relate. In spite of the higher variance, the estimated kinship coefficients show very high correlation between the fully typed (~1.2M high quality diploid markers) diploid and the corrected coefficients of the partially typed diploid and haploid datasets (R=0.9998 and R=0.9991 respectively) compared to the correlation with the uncorrected estimates (R=0.749 and R=0.751; Additional File 5: Table S4) proving that deviations of estimated coefficients from the expected kinship coefficients are the result of true biological variation.” We found no significant bias due to the reduced coverage: according to our simulations regardless of the coverage, we got the same corrected kinship coefficient from the reduced marker haploid data as we from the diploid fully typed data set (Additional File 2: Table S1, Figure 1B). The high correlation between the calculated kinship coefficients from the depleted haploid and fully typed diploid experimental data (Additional File 5: Table S4) also demonstrates this. This means that the observed experimental bias is likely caused by some estimator in the original PC-Relate method.*

*Accordingly, we noted these findings in the Results: Section: The effect of overlapping marker fraction on the kinship coefficient calculation “Although the marker overlap fraction correction differs more than one magnitude between very low and high marker overlap fraction sample pairs, the analysis shows (Additional File 2: Table S1) that the correction itself does not introduce overall bias (the mean is statistically the same) and does not significantly multiply the error rate (4x increase in SD at 20x correction factor). Our results suggests that the increased SD of the method are likely due to the higher uncertainty of PCA.”*

*Section: Statistical validation, assessment of technical errors “In spite of the higher variance, the estimated kinship coefficients show very high correlation between the fully typed (~1.2M high quality diploid markers) diploid and the corrected coefficients of the partially typed diploid and haploid datasets (R=0.9998 and R=0.9993 respectively) compared to the correlation with the uncorrected estimates (R=0.749 and R=0.751; Additional File 5: Table S4). Our result suggests that the applied pseudo-haploidization and correction in the marker depleted experimental data does not introduce overall bias.”*

*and also modified on our conclusion in the Discussion: “However, this downward bias is also present in the kinship coefficient estimation of fully typed diploid 1KG relatives suggesting that the original PC-Relate algorithm and not the applied correction or pseudo-haploidization is accountable for this bias. This is also supported by our simulations on the corrected kinship coefficient calculated from marker depleted pseudo-haploid data and the original fully typed diploid data (Additional File 2: Table S1, Figure 1B) and the very high correlation between the kinship coefficient calculation of marker depleted pseudo-haploidized and the original fully typed 1KG data (Additional File 5: Table S4).”*

- Thank you for clarifying the text regarding READ missing only one relation, etc. This is worth adding to the paper as others may similarly be confused by the current wording.

*Response: We changed the wording in this paragraph to clearly show that this comparison refers to only the READ validated relatives and not the CWC family (at the previous paragraph): „We selected the top 10 populations with the highest number of individuals and performed the READ analysis separately. READ identified relatives up to the 2nd degree. In the selected populations READ identified no additional relatives compared to our methodology. For each identified relatives the degree of kinship was matching between the two methods. In this comparison our method indicated one additional 2nd degree relation (AITI_95_d and AITI_98) that was missed by READ.”*
